# Sulfur–Oxygen Competitive Coordination Allosteric Hydrogel for Triboelectric Nanogenerator‐Based Intelligent Tactile Recognition

**DOI:** 10.1002/advs.77707

**Published:** 2026-09-27

**Authors:** Shixia Lan, Lin Zhang, Yunyun Dong, Yongyun Mao, Wanbiao Hu

**Affiliations:** ^1^ Yunnan Key Laboratory of Electromagnetic Materials and Devices School of Materials and Energy Yunnan University Kunming People's Republic of China; ^2^ Southwest United Graduate School Kunming People's Republic of China; ^3^ School of Software and AI Yunnan University Kunming People's Republic of China; ^4^ School of Engineering Yunnan University Kunming People's Republic of China

**Keywords:** dynamic coordination hydrogel, electronic skin (e‑skin), material recognition, smart glove, triboelectric nanogenerator (TENG)

## Abstract

Flexible sensing technologies based on triboelectric nanogenerators (TENG) have shown promise for wearable electronics and human‑machine interaction. However, existing hydrogel‑based TENGs suffer from an inherent trade‑off between interfacial adhesion and electrical output, and their material recognition capability is compromised in real‑wearing environments due to signal variations. Here, the authors propose a smart glove system integrating a dynamic Ag^+^─S coordinated hydrogel with a machine‑learning‑based material recognition framework. Inspired by biological allostery, a “sulfur‑oxygen competitive coordination” switch is designed using Ag^+^ and a bifunctional small molecule, 5‐carboxythiophene‐2‐boronic acid (CTBA). By adjusting the Ag^+^/CTBA ratio, the hydrogel reversibly switches between a soft, highly adhesive state and a stiff, mechanically robust state. This enables precise modulation of mechanical, adhesive, and triboelectric properties. A lightweight 1D convolutional neural network (WaveformCNN) is developed to classify contact waveforms. The trained model achieves 99.70% accuracy on laboratory‑collected data and 93.35% on real‑wear glove data, demonstrating cross‑scenario generalization. The hydrogel exhibits graded signal output under different finger bending angles and independent five‑finger recognition capability. This work establishes a strategy that bridges molecular coordination design and intelligent tactile perception, offering a new paradigm for self‐powered electronic skins, autonomous material identification, and adaptive human–environment interaction in complex scenarios.

## Graphical abstract

A dynamic Ag^+^─S coordinated hydrogel with a sulfur–oxygen competitive coordination allosteric switch enables reversible tuning between soft–adhesive and stiff–robust states. Integrated with a lightweight 1DCNN classifier, the smart glove system achieves 99.70% laboratory and 93.35% real‐wear material recognition accuracy, offering a paradigm for self‐powered tactile perception and intelligent human–machine interaction.

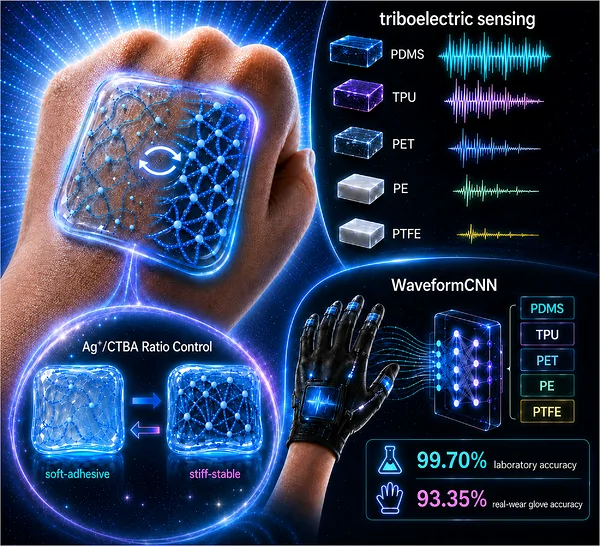

## 1 Introduction

The rapid advancement of flexible sensing technology has greatly promoted the application of wearable electronic devices in fields such as motion monitoring, human‑machine interaction, and intelligent robotics [1, 2]. Among them, triboelectric nanogenerator (TENG)‑based intelligent sensing systems have emerged as highly promising perception platforms due to their self‑powered operation, wide material availability, and fast signal response [3, 4]. In particular, combining TENG with flexible hydrogels not only enables conformal attachment, high stretchability, and biocompatibility, but also generates abundant electrical signals through contact electrification, which can be used to recognize different materials [5]. This material recognition capability is of great value in scenarios such as electronic skin, intelligent prosthetics, and automated sorting [6]. However, existing hydrogel‑based TENGs still face two key challenges in practical applications: (1) The interfacial adhesion and electrical output performance of hydrogels are often difficult to balance, leading to unstable signals during dynamic contact; (2) In real‑wearing environments (e.g., during use of a sensing glove), due to interferences such as contact angle, pressure, speed, and environmental noise, the raw electrical signal differences among different materials become significantly smaller, making accurate material classification impossible using only traditional threshold comparison or simple feature extraction [7]. Therefore, developing a comprehensive strategy that simultaneously optimizes the mechanical and interfacial adhesion properties of hydrogels through molecular design and extracts discriminative features from complex waveforms using advanced machine learning algorithms has become an urgent need to promote the practical application of intelligent tactile recognition technology.

In recent years, considerable efforts have been devoted to improving the overall performance of hydrogel‑based TENGs, including constructing conductive networks, designing self‑healing interfaces, and engineering microstructures [8, 9]. For example, conductive fillers such as polyaniline, carbon nanotubes, or MXene have been introduced to increase the conductivity of hydrogels [10]; dynamic borate ester bonds, hydrogen bonds, or metal‑coordination bonds have been exploited to impart self‑healing ability and extend device lifetime; and micro/nanostructures have been fabricated on the triboelectric layer to enhance contact electrification efficiency [3, 11, 12]. Nevertheless, existing hydrogel materials often suffer from an inherent trade‑off between mechanical strength and interfacial adhesion. High crosslinking density can enhance strength but reduces chain mobility and surface adaptability, leading to poor interfacial adhesion. Conversely, an overly soft network can achieve conformal contact but struggles to provide stable electrical output and fatigue resistance. More importantly, the mechanical and adhesive behavior of most hydrogels is determined by static crosslinking networks, lacking the ability to reversibly switch between different states under external regulation, making it difficult to adapt to the different requirements of “soft‑adhesive” vs “stiff‑stable” states in various contact scenarios [13]. Although metal‑ion coordination (e.g., Fe^3^^+^‑COOH) has been used to construct dynamic networks, it is usually limited to a single coordination mode with a narrow regulation range [14, 15]. Moreover, switching the coordination state often requires external stimuli such as pH changes, light, or temperature, which are not practical for on‑demand regulation in a wearable environment [16]. Therefore, designing a hydrogel system that achieves reversible switching of coordination mode and network topology simply by varying component ratios, while simultaneously optimizing mechanical and interfacial adhesion properties, represents an unsolved but highly valuable direction in this field.

In biology, allostery refers to the binding of an effector at a site distinct from the active site, which induces a conformational change in the protein and thereby remotely modulates its function [17]. Inspired by the allosteric regulation in biological systems, a novel regulatory mechanism based on a “sulfur‑oxygen competitive coordination allosteric switch” was proposed. By using a bifunctional small molecule, 5‐carboxythiophene‐2boronic acid (CTBA), that contains both a sulfur‐containing coordination site (thiophene S) and oxygen‐containing coordination sites (carboxylate O atoms), together with Ag^+^, a competitive coordination system was constructed. Simply by adjusting the molar ratio of Ag^+^/CTBA, precise and reversible switching between a “soft Ag^+^─S coordination” mode and a “rigid Ag^+^─O bridging coordination” mode can be achieved. This design breaks through the limitation of traditional single‑coordination‑mode systems. When Ag^+^:S = 1 (equimolar ratio), the system is dominated by Ag^+^─S coordination; the network crosslinking density decreases, chain mobility increases, and the hydrogel enters a “soft, highly adhesive” state, which facilitates conformal contact with different material surfaces and thereby improves the stability and amplitude of triboelectric signal output. When Ag^+^ is in excess, the coordination mode shifts to Ag^+^─O bridging, forming stable bidentate coordination structures; the network becomes dense again, and mechanical strength and fatigue resistance are significantly restored. This “soft‑stiff” reversible switching not only simultaneously achieves high adhesion and tunable mechanical properties within the same material system, but also induces a topological evolution from a homogeneous network to a microphase‑separated structure enriched with Ag^+^─S coordination domains, further enhancing strain sensitivity and dynamic reconfigurability. To the best of our knowledge, this is the first time that the concept of “competitive coordination allostery” has been introduced into the molecular design of hydrogel‑based TENG, enabling continuous and reversible modulation of interfacial adhesion, mechanical strength, and triboelectric output simply by changing the component ratio.

Based on the above coordination regulation mechanism, the optimized hydrogel is further integrated into a smart sensing glove and used to construct a complete waveform classification and material recognition system. First, in a linear‑motor‑driven laboratory environment, the output signal differences generated by contact between the hydrogel and different materials are obvious and highly distinguishable. In contrast, in the actual wearing environment, due to the randomness of hand movements and inconsistency of interfacial contact, the signal waveforms of different materials become much closer, making direct recognition difficult. To address this, a lightweight 1D convolutional neural network (WaveformCNN) was designed that takes raw voltage sequences as input, extracts local temporal features through three convolutional modules, and then outputs probability distributions for the five material categories via global pooling and a fully connected layer. During model training, a stratified partitioning strategy is used to construct a standardized dataset, and Z‑score normalization is applied to improve convergence. Experimental results show that after training, WaveformCNN achieves 99.70% accuracy on the laboratory test set and still maintains 93.35% accuracy on real‑wear data. In addition, the graded signal output of the hydrogel under different finger bending angles and the capability of independent five‑finger recognition were demonstrated, showcasing the advantages of this intelligent system in continuous strain sensing and multi‑channel decoupling. Unlike traditional metal–ligand dynamic networks that rely solely on a single coordination pair and are incapable of achieving reversible switching of network topology, the proposed “sulfur–oxygen competitive coordination allosteric switch” enables reversible soft–stiff state switching driven by coordination mode, coordination‐induced microphase‐separated topological transition, and synergistic decoupling regulation of mechanical, adhesive, and triboelectric output properties. The proposed Ag^+^─S coordinated allosteric hydrogel not only provides a new model for studying dynamic coordination networks but also opens a new avenue for tactile intelligence and autonomous material recognition of wearable electronic devices in complex scenarios.

## 2 Results and Discussion

### 2.1 Dynamic CTBA/Ag^+^‐Regulated PCA Hydrogel for Flexible Adhesive Sensing Platforms

**FIGURE 1:**
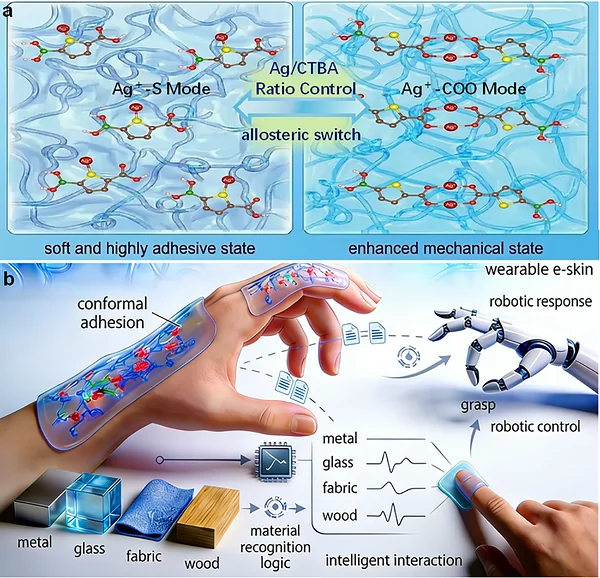
(a) Schematic illustration of the CTBA/Ag^+^‐regulated PCA hydrogel, showcasing its dynamic network architecture with reversibly tunable mechanical strength and interfacial adhesion properties; (b) Application of the PCA hydrogel as a flexible adhesive sensing platform for human motion monitoring and intelligent interactive recognition.

As shown in Figure 1a, this work designs and fabricates a PCA hydrogel based on the regulatory mechanism of a “sulfur–oxygen competitive coordination allosteric switch”. The core of this mechanism lies in introducing the chemical concept of “allostery” into supramolecular systems: using Ag^+^ as the central ion, a typical hard and soft acids and bases (HSAB) competitive system is established between Ag^+^ and 5‐carboxythiophene‐2‐boronic acid (CTBA), which contains both a sulfur‐containing coordination site (thiophene S) and oxygen‐15–containing coordination sites (carboxylate O atoms). By adjusting the ratio of Ag^+^ to CTBA, the coordination configuration can be precisely switched between “soft sulfur‑philic (Ag^+^─S)” and “hard oxygen‑philic (Ag^+^─O)” modes (Figure S1). This transition in coordination mode resembles “allosteric regulation” at the molecular level, directly inducing topological reconstruction of the polymer network's crosslinking density, thereby providing a molecular basis for precisely controlling the mechanical properties and interfacial adhesion behavior of the hydrogel. This hydrogel is synergistically constructed from a polyacrylamide (PAM) polymer network, a multifunctional small‐molecule regulatory unit CTBA, and silver ions (Ag^+^). Specifically, PAM forms a three‐dimensional (3D) continuous polymer skeleton, whose network stability mainly originates from hydrogen bonding interactions among amide groups [18]; CTBA is introduced into the network as a multifunctional regulatory unit, providing abundant coordination and hydrogen bonding sites. Upon introduction of Ag^+^, dynamic coordination interactions are established with CTBA, namely coordination with the sulfur atom on the thiophene ring or bridging structures coordinated with the carboxylate group, laying the molecular foundation for the “sulfur–oxygen competitive coordination allostery” mechanism. Under this mechanistic framework, by adjusting the relative ratio of CTBA to Ag^+^, the mechanical properties and adhesion behavior of the PCA hydrogel can be precisely and reversibly regulated (Figure 1a).

When the molar ratio of Ag^+^ to CTBA approaches the equimolar state, the coordination equilibrium shifts toward the Ag^+^─S side, and this coordination mode becomes dominant (Figure S1a). This consumes some of the CTBA molecules originally involved in network reinforcement, weakening the effective hydrogen bonding interactions between PAM chains, thereby softening the network structure and significantly enhancing interfacial adhesion, transitioning the hydrogel into a soft and highly adhesive state. When Ag^+^ is in excess and the ratio deviates from the equimolar state, the system transitions to the Ag^+^‐COO mode, where Ag^+^ ions redirect coordination to carboxylate groups of CTBA (Figure S1b). This rearrangement generates stronger, more stable crosslinks, significantly elevating network density and enhancing mechanical strength, toughness, and structural stability. Upon further reintroduction of CTBA, the coordination shifts back toward the Ag^+^─S side. CTBA molecules re‐coordinate with Ag^+^, increasing the proportion of Ag^+^─S coordination and inducing the formation of local coordination clusters, thereby once again weakening the effective crosslinking of the PAM network and returning the hydrogel to a soft and highly adhesive state. Through this process, the PCA hydrogel achieves a reversible cyclic transition of “stiffening–softening–restiffening–resoftening” under the precise regulation of the “sulfur–oxygen competitive coordination allostery” mechanism (Figure S2). Benefiting from the reversible mechanical and adhesive properties endowed by this mechanism, the PCA hydrogel functions as a wearable electronic skin (e‐skin) for precise human motion detection. Conformal attachment to skin (e.g., wrist, finger) allows real‐time capture of subtle mechanical stimuli, converting physiological movements into quantifiable electrical signals (Figure 1b). Beyond motion monitoring, the hydrogel exhibits intelligent material recognition capability via characteristic electrical waveform responses. Integrated with robotic control systems, this material recognition enables adaptive grasping strategies—such as modulated contact force for fragile substrates—facilitating seamless human‐machine interaction.

### 2.2 Coordination Mechanism and Spectroscopic Characterization

**FIGURE 2:**
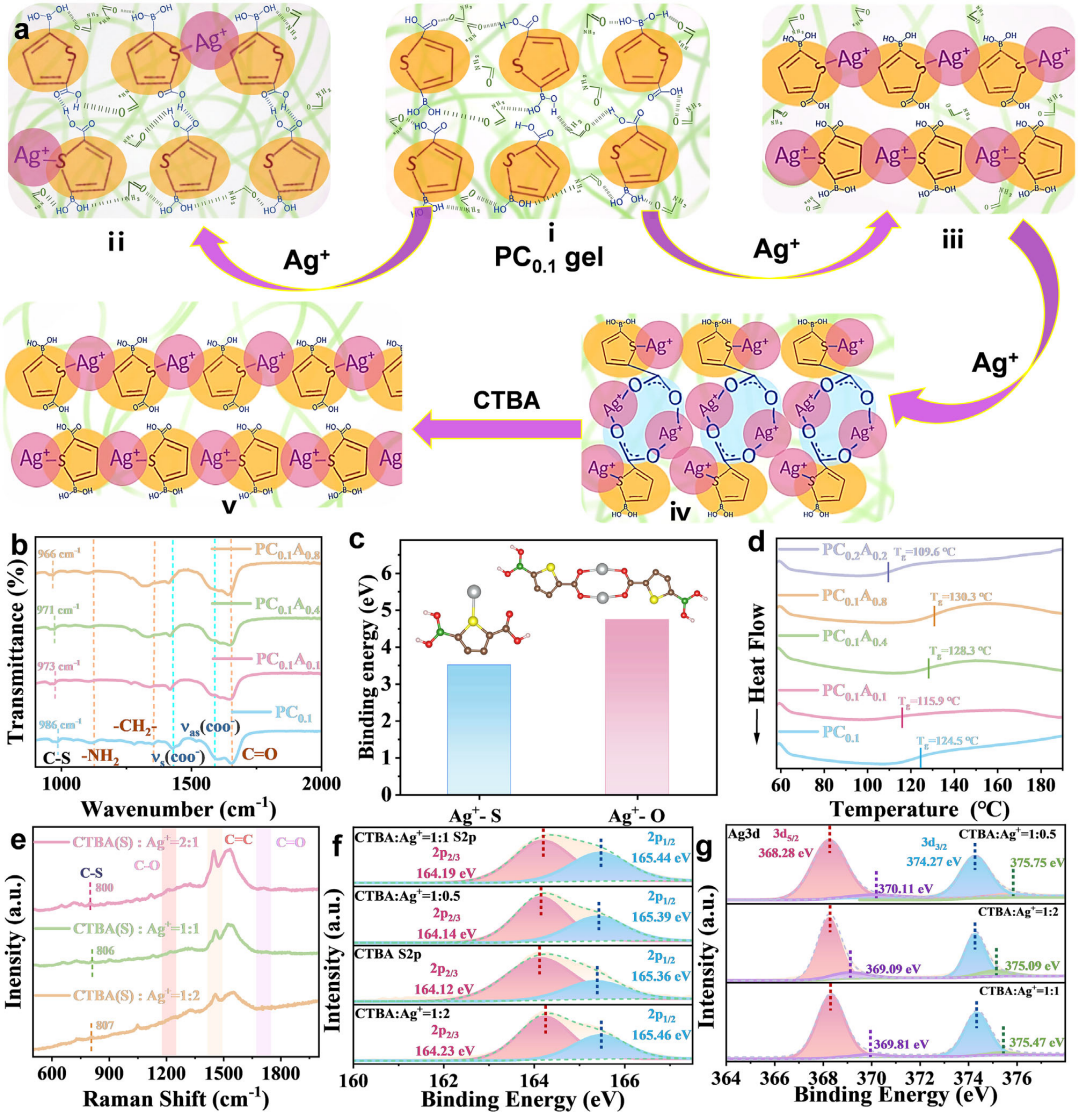
(a) Schematic illustration of the Ag^+^ coordination mechanism within the CTBA/Ag^+^‐regulated PCA hydrogel network. (b) FTIR spectra of the pristine PAM hydrogel (PC) and PCA hydrogels with different Ag^+^ contents. (c) Proposed bonding configurations and calculated binding energies of Ag^+^ coordinated with different functional groups in CTBA. (d) Glass transition temperatures (*T_g_*) of the PC and PCA hydrogels. (e) Raman spectra of pristine CTBA and CTBA with different Ag^+^ doping ratios. (f–g) High‐resolution XPS spectra of the S 2p and Ag 3d regions for pristine CTBA and Ag^+^‐doped CTBA with different CTBA/Ag^+^ ratios.

In the gel network, PAM serves as the continuous polymer backbone, forming a basic 3D network structure through hydrogen bonding between amide groups. The functional regulatory unit, CTBA, is embedded within the PAM network. The molecular structure of CTBA contains both carboxyl and sulfur‐bearing groups, thereby providing a multisite, tunable coordination environment for Ag^+^ ions (Figure 2a‐i). Upon the introduction of Ag^+^, the Ag^+^ ions (4d^10^5s^0^) interact with the thiophene aromatic rings through π‐complexation [19, 20]. In this mechanism, the Ag^+^ can utilize its empty 5s orbital to form a conventional σ bond with thiophene, while its 4d^10^ orbital can back‐donate electrons to the antibonding π* orbital of the sulfur heterocycle. Consequently, thiophene molecules can form coordination structures through σ–π interactions with Ag^+^ (as illustrated in Figure 2a‐ii–iii) [19, 20, 21]. As the Ag^+^/CTBA ratio varies, the relative contributions of the different coordination modes within the network change, inducing a transition between a coordination‐reconfigured state and a dispersed reinforced state. As is evident from Figure 2a‐iv and Figure S2, the PCA hydrogel is not dominated by a single coordination mode. Instead, dynamic network reconstruction is achieved through the synergistic interplay of tunable Ag^+^─S coordination and Ag^+^─O coordination. This multi‐modal coordination mechanism establishes the molecular foundation for the reversible modulation of the mechanical properties and interfacial adhesion behavior of the PCA hydrogel.

Figure 2b and Figure S3 present the FTIR characterization results, which first analyze the changes in functional group interactions within the hydrogel upon Ag^+^ introduction. Compared to the PC_0.1_ hydrogel, PCA hydrogels with varying Ag^+^ contents exhibit distinct differences across multiple characteristic vibrational regions (Table S1). Notably, in the vibrational region below 1000 cm^−1^, associated with sulfur‐containing groups, the peak positions shift with increasing Ag^+^ content, indicating the involvement of sulfur atoms in coordination with Ag^+^ [22]. In contrast, the peak positions of the symmetric and asymmetric stretching vibrations related to carboxyl groups show relatively limited changes, suggesting that the Ag^+^─O coordination structure involving carboxylates remains relatively stable across the studied ratio range [23, 24]. To further elucidate the affinity of Ag^+^ for the different coordinating groups in CTBA, density functional theory (DFT) calculations were employed to determine the binding energies of the Ag^+^─S and Ag^+^─O coordination configurations (Figure 2c). The results show that the Ag^+^─S coordination has a lower binding energy, indicating that this coordination mode is more readily activated and involved in network regulation across different Ag^+^/CTBA ratios. Conversely, the Ag^+^─O coordination exhibits a higher binding energy, suggesting that under limited Ag^+^ conditions, Ag^+^ preferentially coordinates with S, leading to the formation of a soft, adhesive PCA gel. Differential scanning calorimetry (DSC) was further utilized to characterize the influence of Ag^+^ coordination behavior on the thermodynamic properties of the hydrogel (Figure 2d). Compared to the PC_0.1_ hydrogel, the glass transition temperature (*T_g_*) of the PCA hydrogel shows a clear trend with varying Ag^+^ content. When the Ag^+^/CTBA ratio approaches equimolar conditions, the coordination‐driven network reconstruction is dominated by the “soft sulfur‐philic (Ag^+^─S)” coordination configuration, which significantly increases segmental mobility, leading to a decrease in *T_g_* (PC_0.1_A_0.1_) (Figure 2d). This is consistent with the macroscopic behavior of the hydrogel, which exhibits a soft, highly adhesive state. As the Ag^+^ content becomes excessive, the coordination configuration gradually shifts to a “hard oxygen‐philic (Ag^+^─O)” dominated mode, forming bridging bidentate ligands, and *T_g_* gradually increases (PC_0.1_A_0.4_ and PC_0.1_A_0.8_). Upon the reintroduction of CTBA, more thiophene rings coordinate with Ag^+^, weakening the polymer network again, and *T_g_* decreases correspondingly (PC_0.2_A_0.2_) [25, 26].

Raman spectroscopy further revealed the regulatory effect of Ag^+^ coordination on the vibrational characteristics of CTBA molecules (Figure 2e). Upon Ag^+^ introduction, the C─S bond vibration in CTBA undergoes a clear shift from 800 to 807 cm^−1^, accompanied by a weakening of the C═C bond intensity, reflecting changes in the local electronic environment of the sulfur atom [18]. This change becomes more pronounced with increasing Ag^+^ content, indicating a progressively enhanced contribution from Ag^+^─S coordination interactions [20, 27]. In the S 2p core‐level spectra of X‐ray photoelectron spectroscopy (XPS) (Figure 2f), it is observed that as Ag^+^ content increases, the overall peak positions shift to higher binding energies, indicating a decrease in the electron cloud density around the sulfur atoms, consistent with their coordination to Ag^+^. Concurrently, the binding energy of Ag^+^ in the Ag 3d spectra gradually decreases, reflecting enhanced electron donation from the ligands to Ag^+^ during the coordination process (Figure 2g) [28, 29]. The synergistic evolution of the S 2p and Ag 3d core levels clearly reveals the increasing strength of the Ag^+^─S coordination interaction with changes in the Ag^+^/CTBA ratio. These multi‐faceted characterization results consistently demonstrate that the tunable Ag^+^─S coordination interaction formed between Ag^+^ and CTBA within the PCA hydrogel plays a crucial regulatory role in network reconfiguration and local clustering, providing the molecular‐level foundation for the reversible modulation of the mechanical properties and adhesion behavior of the PCA hydrogel.

### 2.3 XAFS Analysis and Coordination‑Induced Phase Separation

**FIGURE 3:**
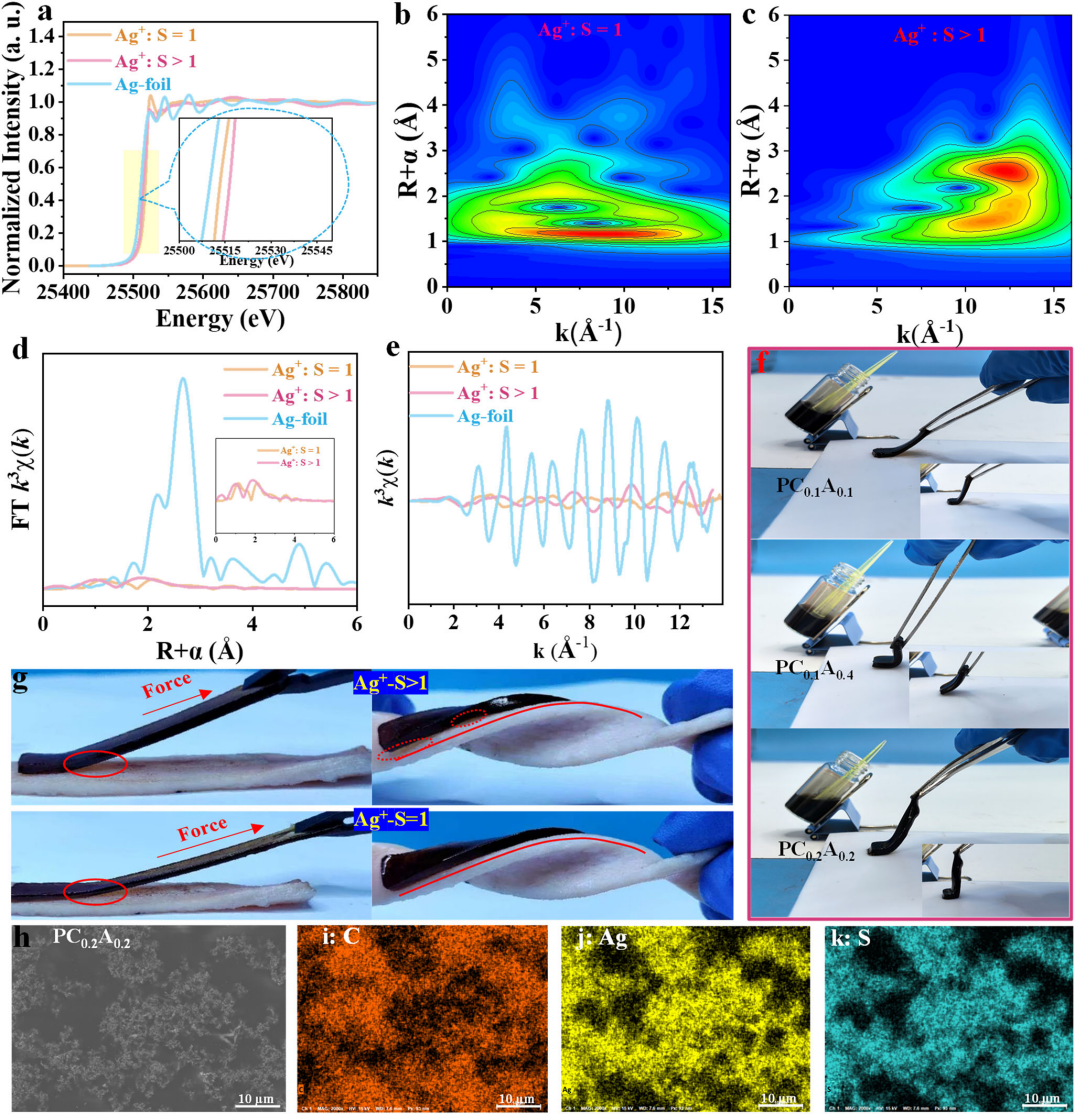
(a) Ag K‐edge XANES spectra of the hydrogels with different Ag^+^─S coordination ratios. (b‐c) Wavelet transform plots for the samples with different Ag^+^─S coordination ratios. (d) Fourier‐transformed EXAFS spectra in R‐space, showing the radial distribution function around Ag atoms. (e) *k*‐space EXAFS spectra of the samples with different Ag^+^─S coordination ratios. (f) Photographs of the hydrogels with different Ag^+^─S coordination ratios, showing the macroscopic structural evolution of the gels. (g) Photographs comparing the interfacial contact behavior of PCA hydrogels at different Ag^+^/S ratios on moist porcine skin under twisting deformations: Ag^+^─S >1 and Ag^+^─S = 1. (h–k) Elemental mapping of PC_0.2_A_0.2_ hydrogel showing the phase separation induced by increased Ag^+^─S coordination content.

To further elucidate the coordination evolution of Ag^+^ at a more detailed electronic structure level, we systematically performed K‐edge X‐ray absorption spectroscopy (XAS) analysis on two representative systems: Ag^+^:S = 1 and Ag^+^:S > 1. As shown in Figure 3a, comparison with the absorption edge of Ag foil reveals that the absorption edges of both coordination samples are significantly higher than that of the metallic silver standard, indicating that silver predominantly remains in the +1 oxidation state without being reduced to Ag(0) [30]. Notably, compared with the Ag^+^:S = 1 system, the absorption edge of the Ag^+^:S >1 sample exhibits a slight shift toward higher energy. This shift is associated with an increase in the local electron cloud density around the central metal atom, suggesting that under higher coordination density, enhanced electron donation from the ligands to Ag^+^ induces a redistribution of the electron cloud around Ag^+^ without altering its oxidation state [30, 31]. Distinct differences are also observed in the white‐line peaks of the near‐edge structure. The Ag^+^:S = 1 system displays a sharp and well‐defined white‐line peak, whereas the Ag^+^:S >1 system shows considerable peak broadening and a slight decrease in intensity (Figure 3a). Given that the white‐line intensity is closely related to the unoccupied electronic state density of the absorbing atom, a decrease in intensity typically implies partial filling of empty orbitals or redistribution of electron density, indicating that upon sulfur coordination, the electron‐donating effect to silver is enhanced, thereby reducing the unoccupied state density at the silver center [30].

Further analysis was conducted using wavelet transform (WT) of extended X‐ray absorption fine structure (EXAFS) spectra (Figure 3b,c and Figure S4a,b). For the Ag^+^:S = 1 sample (Figure 3b and Figure S4c), the WT plot exhibits a single, concentrated intensity distribution, primarily confined to a radial distance range of approximately 1.8–2.5 Å (Table S2), with signals mainly located in the low‑*k* region. Such characteristics typically correspond to a single dominant scattering path involving relatively heavy scatterers, consistent with Ag^+^─S coordination [32]. The elliptical intensity distribution in the plot indicates a relatively simple and homogeneous local coordination environment. In contrast, the Ag^+^:S >1 sample (Figure 3c) shows a significantly broadened intensity distribution extending toward higher *k* regions, with the radial distance expanding to approximately 1.8–3.5 Å (Table S2). The emergence of new intensity features in the high‑*k* region generally suggests the presence of lighter scatterers or multiple scattering paths, consistent with a coexisting coordination environment involving both Ag^+^─S and Ag^+^─O interactions, indicating the formation of a more complex and diverse local structure around silver under high coordination ratios [32].

The Fourier transform results in R‑space (Figure 3d) and the corresponding fitting results (Figure S4a,d) further corroborate the above conclusions. For the Ag^+^:S = 1 sample, two Ag^+^─S coordination shells are fitted with bond lengths of 2.12 and 2.60 Å, respectively, and no typical Ag─Ag coordination is observed (Figure S4a). In contrast, the Ag^+^:S > 1 sample exhibits an enhanced and significantly broadened first‑shell peak, indicating the superposition of multiple coordination paths and a more dispersed bond length distribution (Figure S4d). According to the fitting results shown in Figure S4d, a prominent peak appears at approximately 2.59 Å, corresponding to the typical Ag^+^─S bond length, while a feature near 2.03 Å corresponds to the bridging Ag^+^─O coordination path. Moreover, the EXAFS oscillations (Figure 3e and Figure S4b,e) reveal that the Ag^+^:S = 1 sample exhibits relatively smooth, low‑frequency oscillations consistent with scattering dominated by heavier sulfur atoms. In contrast, the Ag^+^:S > 1 sample displays more complex, high‑frequency oscillations in the mid‑to‑high *k* region (Figure S4e). Because lighter atoms (e.g., oxygen) tend to generate higher‑frequency oscillations, this finding further supports the involvement of Ag^+^─O coordination paths. Furthermore, the wavelet transform plots in Figure S4c,f exhibit pronounced features in the low‐*k* region, indicating the presence of coordination with light elements, specifically O and S. In the case of bridging (bidentate) coordination, the scattering contribution from oxygen is expected to be stronger and sharper, as two oxygen atoms simultaneously contribute to the scattering pathway [30]. Thus, the enhanced oxygen scattering observed in the wavelet analysis indirectly supports the existence of a bridging bidentate coordination mode. The increased complexity of the oscillatory features suggests a greater diversity in the types and distances of coordinating atoms around silver. In summary, combining the XANES, WT, R‑space, and *k*‑space EXAFS analyses, it can be concluded that silver maintains its +1 oxidation state throughout the coordination process. As the coordination density increases, the local electron density undergoes significant redistribution, and the coordination environment gradually evolves from a single Ag^+^─S coordination mode to a mixed system comprising both Ag^+^─S and Ag^+^─O coordination. Concurrently, the electron cloud density around the Ag^+^ center is redistributed, and metal–ligand bridging interactions are enhanced, thereby modulating the internal network structure.

To investigate how this atomic‑scale evolution translates to the macroscopic structural level, we performed a comparative macroscopic analysis of hydrogels with different coordination ratios. When CTBA or Ag^+^ was added alone (Figure S5a–c), the system remained transparent and clear, indicating that both components existed in a molecularly dispersed state and the network maintained a homogeneous single‑phase structure. However, when both components were present in equimolar ratio (e.g., PC_0.1_A_0.1_, Figure S5d,e), the system rapidly developed noticeable turbidity. This turbidity arises from enhanced light scattering due to the formation of coordination‑rich domains, indicating a transition from a homogeneous network to a coordination‑driven microphase‑separated state [33]. At this stage, network rearrangement occurs via Ag^+^─S coordination, leading to a two‑phase structure comprising locally coordination‐rich phases coexisting with a continuous polymer phase. Upon further increasing the silver content (Figure 3f), the PC_0.1_A_0.4_ hydrogel regained mechanical strength, suggesting that Ag^+^─O bridging enhances network robustness. When the Ag^+^‑to‑S ratio was synchronously increased to PC_0.2_A_0.2_, turbidity intensified significantly, and sedimentation stratification occurred upon standing (Figure S5e). This indicates that coordination‑rich domains further grow, and the system gradually evolves from microphase separation to macroscopic phase separation, accompanied by gel collapse, implying that metal–ligand complexes have transformed from molecular or colloidal aggregates into distinguishable phase domains [34]. Figure 3g compares the interfacial contact behavior of PCA hydrogels on moist porcine skin under twisting deformations, with different Ag^+^/S ratios. When the Ag^+^/S ratio is greater than 1, the excess silver ions lead to excessive coordination crosslinking in the hydrogel network, resulting in a stiff structure. Under twisting deformation, the interface between the hydrogel and the moist porcine skin exhibits obvious detachment and slippage, indicating poor conformability and inadequate adhesion. When the Ag^+^/S ratio equals 1, the coordination crosslinking reaches an equilibrium state. The hydrogel network exhibits moderate mechanical strength and good viscoelasticity. Under the same twisting deformation, this hydrogel maintains tight and conformal contact with the moist porcine skin, showing no interfacial delamination or slippage. To further validate the phase separation behavior induced by enhanced coordination, we performed elemental spatial distribution analysis on the PC_0.2_A_0.2_ system. As shown in Figure 3h–k, compared with the elemental mapping of PAM (Figure S6), the sample exhibits pronounced heterogeneity, with irregular enrichment domains ranging from hundreds of nanometers to micrometers appearing on the surface. Notably, the Ag^+^ and S signals are highly colocalized and display distinct clustered distributions rather than a uniform dispersion. This directly demonstrates that the turbid regions originate from the formation of Ag^+^─S coordination‑enriched structures: as the coordination ratio increases, Ag^+^─S coordination progressively evolves from local coordination structures to mesoscale coordination network clusters, leading to significant spatial compositional segregation and indicating the coexistence of coordination‑rich phases and continuous polymer phases within the system. Thus, silver ion coordination not only alters the local electronic structure but also induces a transition of the network from a homogeneous continuous architecture to a heterogeneous biphasic structure by modulating the strength of metal–ligand bridging interactions. This structural transition directly impacts the effective crosslinking density and segmental mobility of the hydrogel, thereby regulating its mechanical properties [35]. Simultaneously, the redistribution of coordination‑rich phases at the interface modifies the surface energy and interfacial dissipation mechanisms, consequently influencing the adhesion behavior of the hydrogel.

### 2.4 Mechanical and Rheological Properties

**FIGURE 4:**
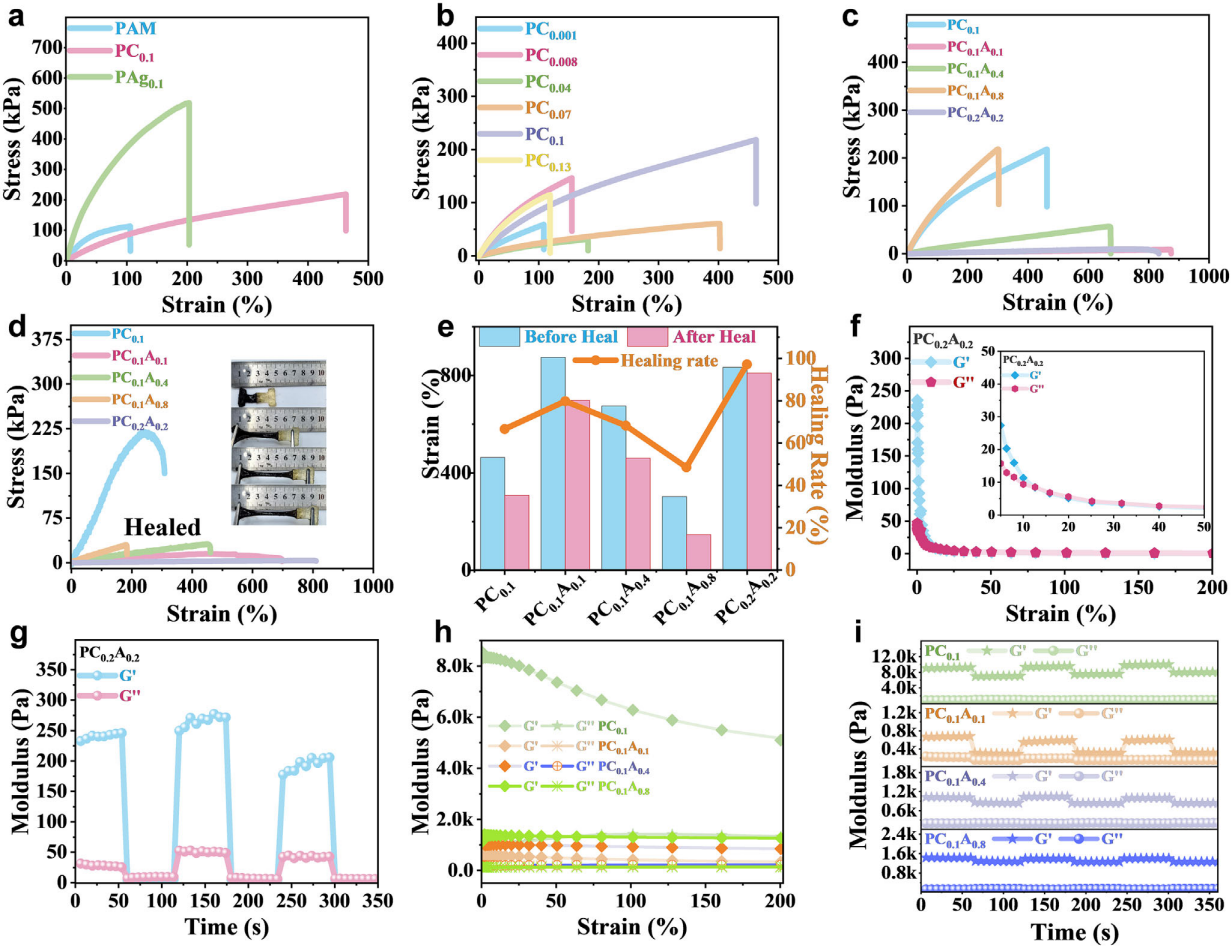
(a) Mechanical properties of PAM, PC_0.1_, PAg_0.1_ hydrogels. (b) Effect of CTBA content on the mechanical properties of PC hydrogels. (c) Stress–strain curves of hydrogels with different coordination ratios. (d) Comparison of tensile stress–strain behavior of the hydrogels after self‐healing, along with a photograph comparing the tensile elongation of PC_0.1_ and PC_0.2_A_0.2_ hydrogels after self‐healing. (e) Self‐healing efficiency and corresponding recovery rate of PC_0.1_ and PCA hydrogels. Rheological strain amplitude sweep curves (storage modulus *G′* and loss modulus *G″*) of (f) PC_0.2_A_0.2_ hydrogel and (h) PC_0.1_ and PC_0.1_A_0.1_/_0.4_/_0.8_ hydrogels. (g) Alternating high–low strain recovery test (*G′* and *G″*) of the PC_0.2_A_0.2_ hydrogel, demonstrating dynamic structural reversibility. (i) Alternating high–low strain recovery test of PC_0.1_ and PC_0.1_A_0.1_/_0.4_/_0.8_ hydrogels, illustrating coordination‐dependent viscoelastic reversibility.

Based on the regulatory mechanism of the “sulfur‐oxygen competitive coordination allosteric switch”, the dynamic coordination induces structural reorganization of the gel network, which also provides an effective means to tune the mechanical and rheological properties of the hydrogel by adjusting its initial composition. To verify this, we first investigated the effect of individual components on the mechanical properties of the gel. As shown in Figure 4a,b, when AgNO_3_ or CTBA was added alone to PAM, the mechanical strength of the gel increased in both cases. The two species existed as fillers within the hydrogel without disrupting the network. According to the Hofmeister effect [36], in a solution containing NO_3_^−^, water molecules are expelled from between polymer chains and hydrogen bonds form among hydroxyl groups, which leads to salting‐in of the polymer, increased solubility, and consequently a decrease in mechanical strength. Therefore, NaNO_3_ and AgNO_3_ were added separately to the PC_0.1_ gel for comparison, and found that the influence of NO_3_^−^ on the mechanical strength was limited (Figure S7). This confirms that the main factor capable of regulating gel strength is the Ag^+^─S dynamic coordination structure. By systematically adjusting the Ag^+^/CTBA coordination ratio, the network structure and viscoelastic equilibrium state can be controllably modulated.

The tensile test results in Figure 4c and Figure S8 clearly show that the coordination ratio has a significant impact on the mechanical properties of the hydrogel. In the PC_0.1_ system, the gel exhibits high tensile strength and moderate elongation at break, indicating that the system is dominated by a stable covalent network. When an equimolar ratio of Ag^+^─S (PC_0.1_A_0.1_) is introduced, the tensile strength markedly decreases while the elongation at break increases significantly (Figure 4c). This suggests that the formed Ag^+^─S dynamic coordination bonds partially disrupt the homogeneity of the original network while simultaneously acting as physical crosslinking points that enhance segmental mobility and energy dissipation [37]. With a further increase in Ag^+^ content (PC_0.1_A_0.4_ and PC_0.1_A_0.8_), the mechanical strength of the system gradually recovers and even improves. This indicates that at high coordination density, Ag^+^─O coordination begins to play a bridging and reinforcing role, and the formation of coordination structures rebuilds a more continuous physical crosslinking network, thereby enhancing overall structural stability. When CTBA is re‑added, the proportion of equimolar Ag^+^─S coordination increases (PC_0.2_A_0.2_), and the mechanical performance of the system decreases again. Combined with the aforementioned phase separation results (Figure 3h), it can be inferred that after coordination‑induced phase separation occurs, the coordination‑rich domains further distribute the stress within the network under applied stress, reducing network strength and increasing the strain capacity of the gel [35]. To further evaluate the effect of different coordination ratios on self‑healing behavior, we performed tensile tests on the healed hydrogels. From Figure 4d and Figure S9, it can be observed that the extent of recovery of tensile properties varies significantly among the samples. Notably, the PC_0.1_A_0.8_ sample could not self‑heal to its initial state after fracture. This is mainly because excess Ag^+^ forms stable bridging bidentate ligand structures with carboxylate groups, resulting in a lack of sufficient reversible crosslinking points in the network and hindering effective reconstruction of the fractured interface [37]. In contrast, samples with a small amount of Ag^+^ all show good recovery of strength and elongation, demonstrating that Ag^+^─S dynamic coordination plays a key role in the interfacial reassembly process. Figure 4e further reveals the criticality of Ag^+^─S dynamic coordination: the equimolar coordination samples (PC_0.1_A_0.1_ and PC_0.2_A_0.2_) exhibit high self‑healing efficiency, indicating that reversible metal–ligand bonds can rapidly reconstruct at the fractured interface. However, when the coordination density is too high (PC_0.1_A_0.8_), the coordination is dominated by stable bridging bidentate ligands between Ag^+^ and carboxylate groups, which effectively enhances network strength but restricts free segmental motion, leading to decreased self‑healing efficiency [38].

Rheological analysis further elucidates the evolution mechanism of the network structure. As shown in Figure 4f, an oscillatory strain sweep test was performed on the PC_0.2_A_0.2_ hydrogel. In the low‑strain region, the storage modulus *G′* of the gel is approximately 250 Pa, significantly higher than the loss modulus *G″* (about 50 Pa), indicating that the system is predominantly elastic and exhibits relatively stable gel‑reinforced characteristics [39]. As the oscillatory strain increases, *G′* rapidly drops below 30 Pa, and *G′′* also gradually decreases. When the strain exceeds 15%, *G′* < *G′′*, and the system becomes dominated by viscous dissipation; the network has entered a state with enhanced fluidity where the softened state prevails [40]. The overall strength is low, and the segments and dynamic interaction sites are more prone to rearrangement under external force, thereby providing good interfacial recovery ability [41]. Figure 4g shows the strain cycling test results for PC_0.2_A_0.2_. Under low‑strain conditions, the gel network maintains an intact elastic structure. When a high strain is applied, *G′* rapidly decreases, transitioning from elasticity‑dominated to viscosity‑dominated behavior [37]. However, when the strain returns to a low level, *G′* quickly recovers, and *G″* also returns to near its initial level; the gel reverts to elasticity‑dominated behavior, and this process can be repeated over multiple cycles, indicating that the gel has good structural recovery and dynamic reconfiguration ability under deformation. To further compare the strain‑response behavior of hydrogel networks with different coordination ratios, oscillatory strain sweep tests were performed on each sample. As shown in Figure 4h, the storage modulus *G′* of the different samples exhibits significant differences. Among them, the PC_0.1_ sample shows the highest initial storage modulus, approximately 9 kPa, which decreases slowly with increasing strain and remains much larger than the loss modulus, indicating that a relatively dense and stable network structure has formed inside this system, providing strong resistance to external deformation. In contrast, after the introduction of Ag^+^, the overall modulus of the samples is significantly reduced. PC_0.1_A_0.1_ exhibits a relatively low storage modulus *G′* and a low loss modulus *G″*, indicating that the internal network has become loose, is sensitive to strain, and is in a softened state prone to reconfiguration. However, as the Ag^+^ content increases, the storage moduli of PC_0.1_A_0.4_ and PC_0.1_A_0.8_ recover to approximately 1.2 and 1.5 kPa, respectively, indicating that the network rigidity is re‑enhanced. This trend is consistent with the tensile test results, confirming that different coordination structures significantly affect the overall network stability and deformation resistance. Figure 4i presents the cyclic modulus variation of samples with different coordination ratios under alternating high‑ and low‑strain conditions. For PC_0.1_, PC_0.1_A_0.4_, and PC_0.1_A_0.8_, although the storage modulus *G′* changes accordingly during the high‑low strain switching, *G′* remains higher than *G″* throughout the entire cycle, indicating that the system is elasticity‑dominated. In contrast, PC_0.1_A_0.1_ and PC_0.2_A_0.2_ both exhibit states where *G′* is close to *G″* during the cycling process (Figure 4g,i), indicating that the gel network becomes relaxed and viscosity‑dominated under high strain, overall displaying a softened state. When the strain reaches 15%, the storage modulus *G′* and loss modulus *G″* of PC_0.2_A_0.2_ intersect (Figure 4f), indicating that the phase separation behavior induced by the increased Ag^+^─S coordination content promotes the formation of a heterogeneous structure in which coordination‑rich regions coexist with relatively loose regions. This structure endows PC_0.2_A_0.2_ with higher strain sensitivity and dynamic reconfigurability, not only making it easier to enter the softened state but also imparting superior interfacial adaptability and recovery potential. In comparison, the storage modulus *G′* and loss modulus *G″* of PC_0.1_A_0.1_ are nearly equal at low strain, exhibiting rheological characteristics similar to those of PC_0.2_A_0.2_ (Figure 4h).

### 2.5 Interfacial Adhesion and Triboelectric Output

**FIGURE 5:**
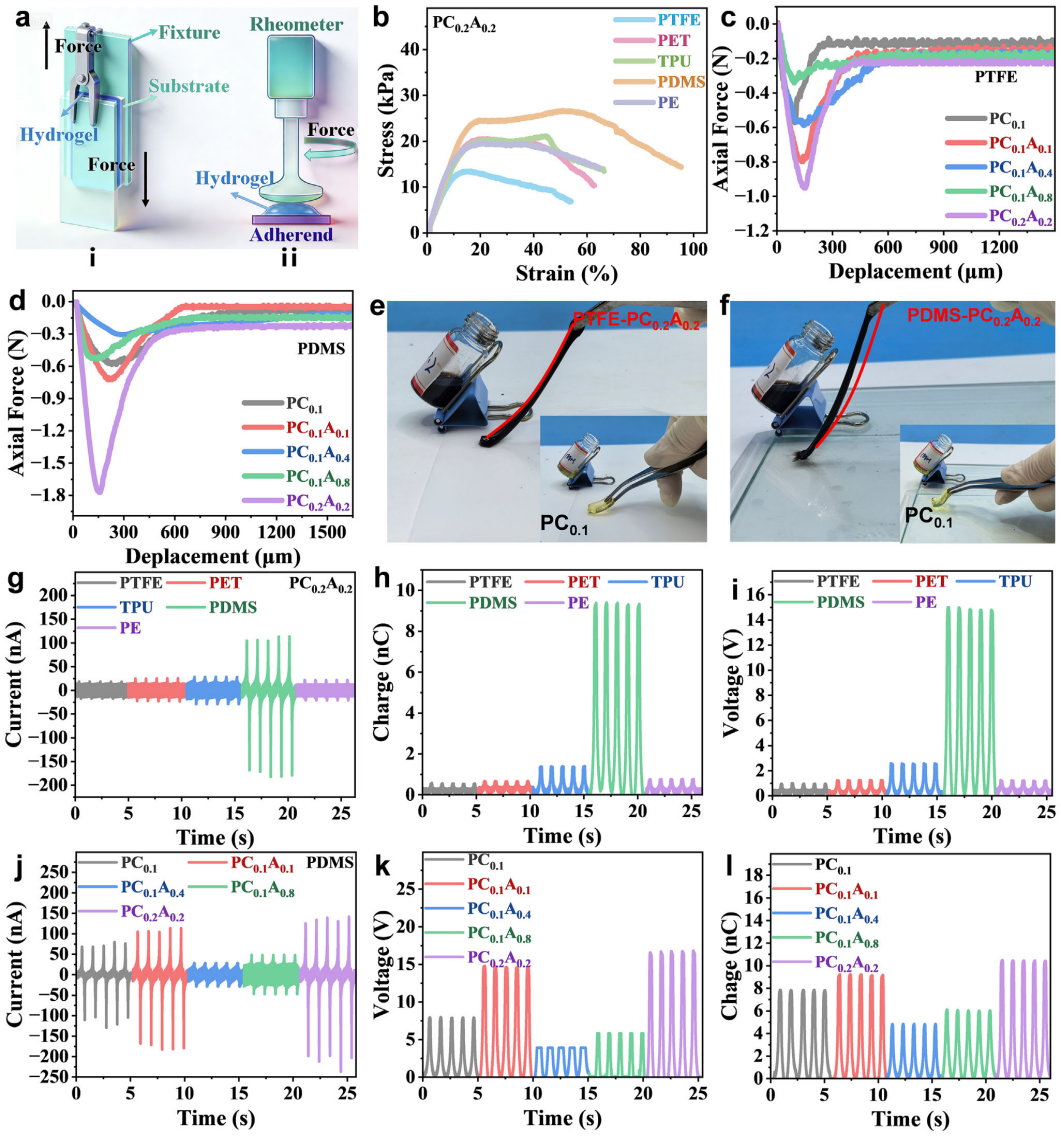
(a) Schematic illustration of the lap‐shear and vertical peel adhesion tests used to evaluate the interfacial interaction between the hydrogel and different substrates. (b) Comparison of the adhesion strength of PC_0.2_A_0.2_ hydrogel on various substrates, including PTFE, PET, TPU, PDMS, and PE. (c–f) Rheometer‐based adhesion measurements of hydrogels with different Ag^+^ contents on PTFE and PDMS substrates, along with representative photographs comparing the adhesion behavior of PC_0.2_A_0.2_ and PC_0.1_. (g–i) Output current, transferred charge, and output voltage of triboelectric devices based on PC_0.2_A_0.2_ hydrogel films paired with different triboelectric counterparts. (j–l) Output current, output voltage, and transferred charge of triboelectric devices constructed from hydrogels with varying Ag^+^ coordination densities against PDMS.

Figure 5a schematically illustrates the two tests used to evaluate the interfacial adhesion performance of the hydrogels: the lap‐shear test and the vertical peel test. In the lap‐shear test, the hydrogel is sandwiched between two substrates to form an overlapping interface. A shear force is applied parallel to the interface, subjecting it to shear stress until slippage or debonding occurs (Figure 5a‐i). In the vertical peel test, after the hydrogel contacts the substrate, a tensile force is applied perpendicular to the interface, inducing normal separation. This test directly reflects the interfacial bonding strength and energy dissipation capability of the gel during contact‑separation cycles (Figure 5a‐ii) [42]. Figure 5b and Figure S10 compare the adhesion performance of the PC_0.2_A_0.2_ hydrogel on different substrate materials (PTFE, PET, TPU, PDMS, and PE). The results show that PC_0.2_A_0.2_ exhibits the strongest adhesion to PDMS and the weakest to PTFE. This difference reflects variations in interfacial energy matching and molecular interaction strength between the Ag^+^─S coordination network and different polymer surfaces. To further investigate the regulatory role of Ag^+^─S coordination on interfacial adhesion behavior, the two materials with the largest adhesion difference (PDMS and PTFE) were selected, and rheological adhesion tests were performed on hydrogels with different Ag^+^ concentrations (Figure 5c,d). The results indicate that when Ag^+^─S coordination moderately loosens the network structure, the viscoelasticity of the hydrogel increases, enhancing its conformal contact ability with the substrate and significantly improving interfacial adhesion. This trend is visually confirmed by photographs of the peeling process (Figure 5e,f): the gel undergoes obvious tensile deformation when peeled from PDMS, whereas the deformation is much smaller on PTFE, indicating stronger interfacial bonding and greater energy dissipation on the former. This behavior demonstrates that under Ag^+^─S coordination, the gel becomes soft and fluid, allowing it to conform more fully to the substrate surface and penetrate microscopic gaps, thereby increasing the real contact area and enhancing interfacial charge and material transfer efficiency [43]. Moreover, the abundant coordination sites and flexible chain segments in the Ag^+^─S coordination system facilitate the formation of interfacial adhesive bonds and promote local charge transfer during bond formation and rupture. In addition, stronger interfacial adhesion contributes to more interfacial fracture and material transfer during contact, generating more charged species and increasing the surface charge density [44]. Thus, Ag^+^─S coordination enhances the interfacial effects of the hydrogel.

Figure 5g–i and Figure S11 present the output current, output voltage, and transferred charge of the PCA hydrogel when in contact with different materials. It can be observed that the device output performance varies significantly with the contact material. When contacting and separating from an electron donor or a material with relatively low electronegativity (e.g., PDMS), the device exhibits higher output performance. In contrast, when contacting a strongly electronegative material (e.g., PTFE), the output signal decreases. Although PTFE has a strong electron‑accepting ability in the triboelectric series [45, 46], its weak interfacial adhesion with the hydrogel limits the real contact area and reduces the interfacial charge transfer efficiency, thereby weakening the overall output performance [47]. Figure 5j–l further compare the output current, voltage, and transferred charge of hydrogels with different Ag^+^ concentrations when contacted and separated from PDMS. As the degree of Ag^+^─S coordination increases (PC_0.1_A_0.1_ and PC_0.2_A_0.2_), the viscoelasticity of the gel network increases, conformal interfacial contact improves, charge generation capability enhances, and the device output performance increases. However, when the Ag^+^ content increases further, the electronic structure of the gel is significantly modulated, and its surface electron‑donating ability changes accordingly. At this stage, device output is no longer dominated by interfacial adhesion strength but gradually becomes governed by the difference in electron affinity between the materials. As the Ag^+^ content increases, the position of the gel in the triboelectric series shifts, and the electron transfer driving force becomes the key factor determining the output signal strength. Thus, Ag^+^─S coordination plays different dominant roles at different ratios: at equimolar ratios, enhanced interfacial adhesion is the main contributing factor; at higher Ag^+^ contents, the difference in electron affinity re‑dominates. The competition and transition between these two mechanisms together determine the final output performance. Figure S12a–c investigate the output voltage, current, and corresponding peak power density of the hydrogel‑based TENG under different external load resistances, as well as the charging curves of capacitors with different capacitances. Figure S12d–i document the practicality of the TENG for energy harvesting and sensing when the gel is attached to different parts of the human body. In summary, Ag^+^─S coordination not only regulates the mechanical and rheological properties of the hydrogel but also directly affects the triboelectric output performance by altering interfacial adhesion behavior, providing a tunable structural design strategy for optimizing hydrogel‑based TENG devices.

### 2.6 Material Recognition and Machine Learning‑Based Smart Glove

**FIGURE 6:**
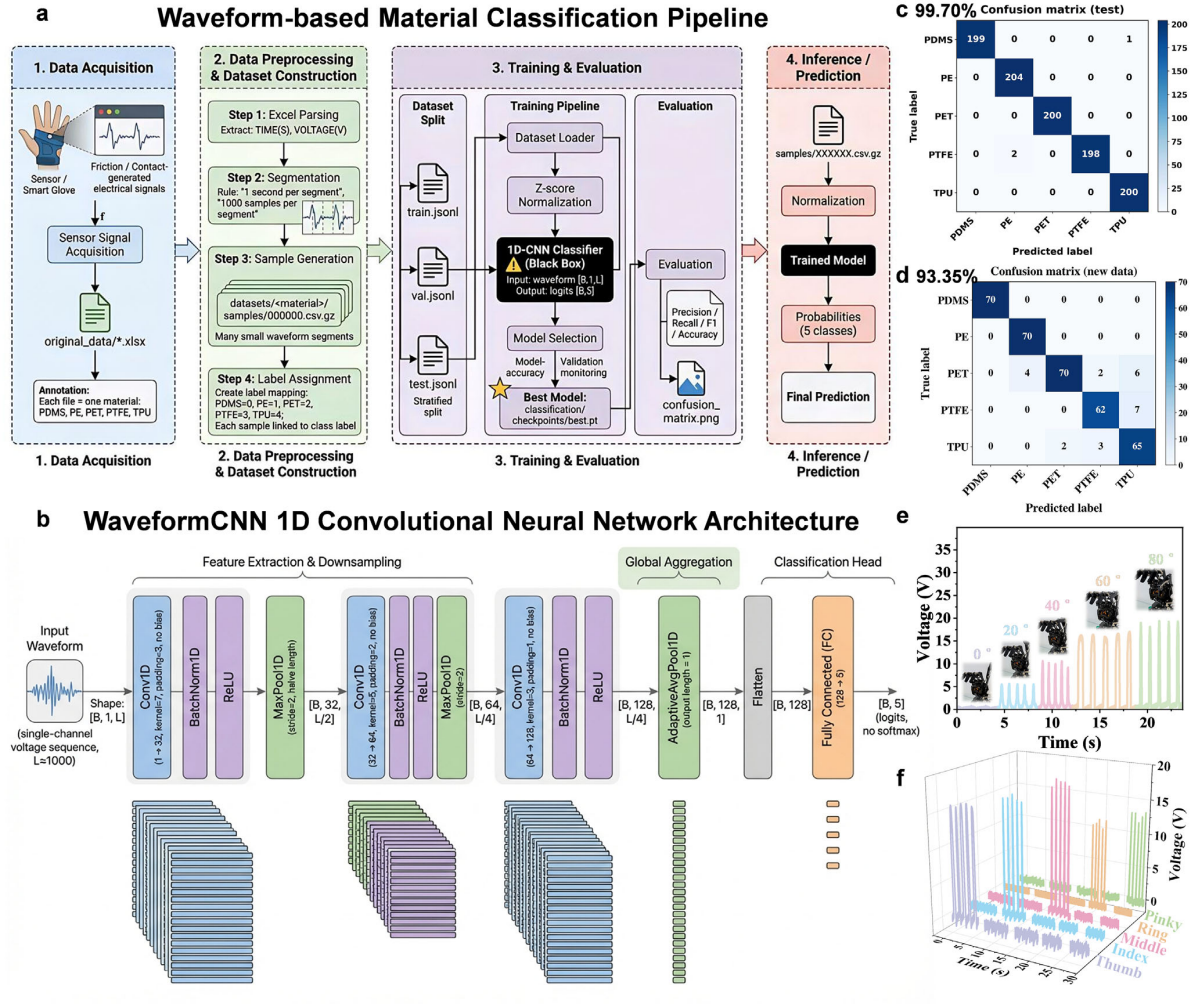
(a) Schematic illustration of the overall waveform‐based material classification framework. (b) Schematic of the WaveformCNN model architecture. (c) Confusion matrix for material classification in the laboratory environment, showing the predictive accuracy for different materials. (d) Confusion matrix for material classification in the real‐wear environment, showing the predictive accuracy for different materials. (e) Output signals corresponding to different finger bending angles (0°, 20°, 40°, 60°, and 80°). (f) Three‐dimensional (3D) output comparison of different fingers (thumb, index, middle, ring, and pinky).

From the triboelectric test results, under the regulation of Ag^+^─S coordination, the hydrogel exhibits significant electrical signal differences when in contact with different materials. Furthermore, integrating the Ag^+^─S coordinated hydrogel into a wearable sensing system endows it with the potential for electronic skin applications, based on which a smart glove and an electronic skin platform were constructed. We first compared the signal output capability of hydrogels with different Ag^+^─S coordination ratios. As shown in Figure S13a, when Ag^+^:S > 1, the output signal amplitude of the hydrogel during finger bending is relatively low. This is because the weak interfacial adhesion and electrical output capability of the hydrogel limit the bending range of the robotic hand. In contrast, Figure S13b demonstrates that under the condition of Ag^+^:S = 1, during finger bending, the Ag^+^─S coordination optimizes the hydrogel network structure, improves interfacial charge transfer efficiency and electrical output capability, and significantly enhances the signal amplitude, thereby enabling a larger bending range of the robotic hand. Figure 6a presents the overall architecture of the hydrogel using a machine learning model for material recognition in different scenarios, illustrating a complete waveform classification framework for material recognition tasks. The specific process is as follows: First, the system collects raw electrical signals generated during contact or friction with different materials via the sensing glove, and saves them as continuous time‐series data in Excel format. In the data preprocessing stage, time and voltage information are extracted from the raw files, and the long continuous waveform is segmented into a large number of short, fixed‐length segments according to predefined rules, thereby generating samples suitable for machine learning processing. At the same time, each sample is assigned a corresponding category label based on the material type, ultimately constructing a standardized supervised learning dataset. In the model training stage, the dataset is divided into training, validation, and test sets using a stratified partitioning strategy to ensure a balanced distribution of samples across categories. Subsequently, feature learning and classification modeling are completed through data loading, Z‐score normalization, and a 1D‐CNN classifier [48]. The model takes 1D waveform sequences as input, outputs prediction scores for each material category, selects the model based on validation set performance, and saves the optimal model parameters. Finally, in the inference stage, new unknown waveform samples are normalized identically to the training stage and fed into the trained model, which outputs the probability distribution of five candidate material categories, and the category with the highest probability is taken as the final prediction. This realizes a complete closed loop from raw signal acquisition, sample construction, model training, to actual material recognition.

The original lightweight 1D convolutional neural network (WaveformCNN) model architecture is shown in Figure 6b. It is a lightweight 1D‐CNN network for 1D waveform signal classification tasks [48]. It is worth noting that the core objective of this study is to validate the feasibility of allosteric hydrogels as triboelectric sensing materials for intelligent recognition. Therefore, a lightweight 1D‐CNN is chosen as the classifier to strike a balance between recognition accuracy and edge‐side deployment efficiency. Its input is a single‐channel voltage sequence of shape ([B, 1, L]), where L ≈ 1000 represents the time‐series length of each sample. The whole network first completes local temporal feature extraction and downsampling through three progressively deepening 1D convolutional modules: the first layer uses 32 convolution kernels to extract basic waveform patterns, followed by BatchNorm1d and ReLU to improve training stability and non‐linear expression capability, and then max pooling compresses the sequence length to L/2. The second layer expands the number of feature channels to 64 and repeats the “convolution—normalization—activation—pooling” structure, gradually elevating the feature representation from shallow local textures to higher‐level temporal structures while reducing the length to L/4. The third layer increases the feature channels to 128, enhancing discriminative ability while retaining key signal variation patterns, resulting in a high‐dimensional feature map of shape ([B, 128, L/4]). Subsequently, the network applies adaptive average pooling to aggregate the temporal dimension to 1, achieving global feature compression and outputting ([B, 128, 1]), which is then flattened into a compact feature vector of ([B, 128]). Finally, a fully connected layer maps it to a 5D output space, obtaining logits scores for five material categories. Overall, the model follows a design philosophy of “local feature extraction—multi‐level abstract representation—global aggregation—linear classification”. It can effectively capture local oscillations, peak changes, and temporal patterns in waveform signals, while controlling parameter size and computational complexity through progressive downsampling and global pooling, thus maintaining strong classification ability with good training efficiency and engineering deployability.

To evaluate the material recognition capability of the hydrogel in different application scenarios, we tested the output signals of its triboelectric nanogenerator in both a linear‐motor‐driven laboratory environment and an actual sensing glove wearing environment. The real‑wear data were collected through multiple contacts performed by the operator in a natural and casual manner, without imposing any constraints on contact force, speed, or angle, so as to introduce signal variations representative of less‐controlled manual‐contact conditions. As shown in Figure 6c,d, in the laboratory environment, the output signal differences corresponding to different materials are relatively obvious, exhibiting good distinguishability. In contrast, in the actual wearing environment, after attaching the hydrogel to the sensing glove, the output signals generated by different materials become much closer (Figure S14). Combined with the laboratory results (Figure 5h), it can be seen that under actual wearing conditions, it is difficult to directly distinguish different materials based solely on raw electrical signals. When the data from both environments were fed into the model without training, the material recognition accuracy of the model was low in both the laboratory and actual wearing environments (Figure S15a,b). This indicates that raw signal differences do not directly translate into effective recognition capability, especially in actual wearing environments where signal differences diminish, making material recognition more dependent on subsequent model training and feature extraction [49]. After training, our proposed WaveformCNN demonstrated strong material classification capability in both machine‐collected data and sensing glove‐measured data. On the standard test set constructed from machine‐collected data, the model achieved an accuracy of 99.70%, with the confusion matrix almost completely distributed along the main diagonal, indicating that under controlled experimental conditions, the model can very accurately distinguish five materials: PDMS, PE, PET, PTFE, and TPU (Figure 6c). Specifically, only a very small number of misclassifications occurred, e.g., 1 PDMS sample misclassified as TPU, and 2 PTFE samples misclassified as PE; the vast majority of samples in other categories were correctly identified. This demonstrates that the model has fully extracted stable and effective discriminative features from the machine‐collected waveform signals. Furthermore, on real new data collected by the smart sensing glove, the model still achieved an accuracy of 93.35%, showing good generalization ability and practical application potential (Figure 6d). Compared with the machine test data, the confusion in the glove test scenario increased somewhat, with errors mainly concentrated among PET, PTFE, and TPU. For example, some PET samples were misclassified as PE, PTFE, or TPU; some PTFE as TPU; and some TPU as PET or PTFE. This indicates that under real manual operation conditions, factors such as contact angle, friction force, motion consistency, environmental noise, and sensor response fluctuations cause a shift in sample distribution compared to the machine test scenario, thereby increasing classification difficulty. Overall, these results demonstrate that the model has near‐perfect recognition performance on machine test data while maintaining high accuracy and robustness on sensing glove‐measured data, validating the effectiveness of the proposed waveform classification framework and providing strong support for its subsequent deployment in real material perception scenarios.

The WaveformCNN model exhibits good cross‐scenario generalization capability, achieving high‐accuracy material recognition under both laboratory test platform and actual wearable sensing glove conditions, indicating that it can adapt to signal variations in different application scenarios while maintaining stable classification performance. In addition, we tested the electrical signal output capability of the hydrogel under different finger bending angles and on different fingers. As shown in Figure 6e, as the bending angle increases, the signal intensity gradually increases, indicating that the hydrogel has excellent strain sensitivity and continuous angle recognition capability. Figure 6f is a 3D mapping of output signals from five fingers (thumb, index, middle, ring, and little finger). The test results show clear separation of signals from each channel, indicating that the sensing glove has the capability of independent multi‐finger recognition. These results demonstrate that the PCA hydrogel sensing material and the constructed intelligent recognition system have good practical application potential and are expected to play a role in smart wearables, human‑environment interaction, and material recognition in complex scenarios [50]. Compared with recently reported AI‐assisted tactile sensing systems, this work demonstrates comparable material recognition performance, achieving accuracies of 99.70% under controlled laboratory conditions and 93.35% under real‐wear conditions, while also demonstrating adhesion tunability and independent multi‐finger recognition [51, 52].

## 3 Conclusion

In conclusion, we present a paradigm‑shifting strategy that integrates molecular coordination design with machine learning for intelligent tactile sensing. The core innovation lies in a “sulfur‑oxygen competitive coordination allosteric switch”, which for the first time enables reversible, composition‑only switching between a soft, highly adhesive state and a stiff, mechanically robust state in a hydrogel‑based TENG. This dynamic regulation not only overcomes the long‑standing trade‑off between interfacial adhesion and electrical output, but also induces a unique microphase‑separated topology that enhances strain sensitivity and reconfigurability. Building on this material platform, we constructed a smart glove system and developed WaveformCNN, a lightweight 1D‑CNN classifier. Remarkably, the trained model achieves 99.70% accuracy under controlled laboratory conditions and retains 93.35% accuracy in real‑wear environments, demonstrating exceptional cross‑scenario generalization—a critical step toward practical deployment. Furthermore, the hydrogel's graded response to finger bending angles and its capability for independent five‑finger recognition highlight its potential for sophisticated human‑machine interaction. This work establishes a paradigm from allosteric coordination engineering to deep‑learning‑enhanced tactile perception, offering transformative opportunities for self‑powered electronic skins, autonomous material identification, and adaptive robotics in unstructured environments.

## 4 Experimental Section

### 4.1 Materials

The chemical reagents used in this study, including acrylamide (AM, AR, 99%), 5‐carboxythiophene‐2‐boronic acid (CTBA, 98%), silver nitrate (AgNO_3_, AR), N,N′‐methylenebisacrylamide (MBAA, 99%), and N‐methylolacrylamide (NMA, 98%), were all purchased from Shanghai Aladdin Biochemical Technology Co., Ltd. Ammonium persulfate (APS) was purchased from Shanghai Macklin Biochemical Technology Co., Ltd. All the reagents were used as received.

### 4.2 Synthesis of PC, PA and PCA Hydrogels

The preparation of PC, PA, and PCA hydrogels is as follows (full formulations are listed in Table S3). A mixed solution containing acrylamide (AM), crosslinkers (NMA and MBAA in varying mass ratios), and CTBA was stirred at 250 rpm and 40°C for 30 min. Ammonium persulfate (APS) was then added as an initiator, and after thorough mixing, the solution was cast into a PTFE mold and crosslinked at 40°C for 2 h to obtain the hydrogel. For the PCA series (denoted PC_x_A_y_, where *x* and *y* are the nominal molar concentrations of CTBA and AgNO_3_), the incorporation of Ag^+^ not only enhances conductivity but also coordinates with the sulfur atom of the thiophene ring, weakening the network crosslinking and yielding a soft, highly adhesive gel. When the Ag^+^ content is further increased, Ag^+^ forms bridging bidentate ligands with the carboxylate groups, increasing steric hindrance and strengthening the gel. Conversely, raising the CTBA content restores Ag^+^–thiophene coordination and reverts the gel to its softened state. Thus, by simply adjusting the CTBA/Ag^+^ ratio, the PCA hydrogel exhibits reversibly tunable mechanical and adhesive properties. The PA hydrogel is prepared following the same procedure but without CTBA (i.e., only AgNO_3_ is added). Detailed compositions are provided in Table S3.

### 4.3 XAFS Measurements and Data Analysis

The X‐ray absorption fine structure (XAFS) measurements were performed at the Shanghai Synchrotron Radiation Facility (SSRF) beamline operating in transmission mode. The liquid sample was sealed in a polytetrafluoroethylene (PTFE) sample cell with Kapton film windows (125 µm thickness) on both sides. The appropriate beamline was selected based on the absorption edge energy (K‐edge or L‐edge) of the target element. Data were collected at room temperature using ionization chambers to monitor the incident (I0) and transmitted (It) beam intensities. Energy calibration was simultaneously performed using metal foil reference samples (e.g., high‐purity copper foil for Cu K‐edge measurements). The energy scan range was set from −200 eV below to 1000 eV above the absorption edge, with an integration time of 1 s per data point and 3 consecutive scans to improve the signal tonoise ratio. Data processing and analysis were performed using the Athena and Artemis modules of the Demeter software package (version 0.9.26). The raw data were first aligned and merged, followed by removal of glitches and outliers. Background subtraction was conducted using the Autobk algorithm, with the normalization energy E0 set at the maximum derivative of the absorption edge. The EXAFS oscillation function *χ*(*k*) was extracted using a cubic spline function, with a *k*‐weight of 3 to ensure evenly weighted oscillations across the *k*‐space range. For Fourier transformation, the *k*‐range was set from 2.5 to 12.5 Å^−1^ using a Kaiser–Bessel window function (d*k* = 1) to obtain the radial structure function. Coordination structure fitting was performed using the Artemis module with nonlinear least‐squares fitting based on scattering paths calculated by FEFF9 theory. To ensure data quality, reference standards were simultaneously measured during the experiment to verify energy calibration accuracy (deviation < 0.5 eV). Data reproducibility was checked through multiple scans, with differences in χ(*k*) signals less than 5%. Principal component analysis (PCA) was employed to verify sample homogeneity and exclude potential beam damage or sample degradation effects. For fluorescence mode data, self‐absorption correction was also applied (using the FLUO algorithm in ATHENA).

### 4.4 Raman Characterization

Raman spectroscopy was performed using an Invia Raman spectrometer (Renishaw, UK). The hydrogel samples (PC_0.1_, PC_0.1_A_0.1_, PC_0.1_A_0.2_, PC_0.1_A_0.4_, and PC_0.1_A_0.8_) were cut into 1 × 1 cm pieces and fixed onto the microscope stage. The microscope focusing system was adjusted to precisely focus the laser spot (excitation wavelength: 532 nm) onto a specific area on the hydrogel surface. Raman signals were acquired after background subtraction and baseline correction using the Raman software. Multiple measurements were taken for each sample to obtain spectra with a high signal‑to‑noise ratio. By comparing the characteristic peaks (O─H, C═C, and COO^−^) in terms of position, intensity, and full width at half maximum (FWHM), the changes in the chemical structure of the hydrogels were analyzed.

### 4.5 Self‐Healing Experiments

The self‐healing after cutting each of the hydrogel samples, they were allowed to heal at the same temperature for 1 h. The strain of the healed samples was then measured. The self‑healing efficiency was quantitatively calculated as the ratio of the strain after healing to the strain before cutting: Self‑healing efficiency (%) = (Strain after healing)/(Strain before cutting).

### 4.6 Rheological Properties Testing

Rheological tests were performed using an HR20 rotational rheometer (TG Company, Germany). (1) Deformation and recovery test under alternating low and high strains: At room temperature and a fixed angular frequency of 10 rad/s, PC_0.1_ and PC_0.1_/_0.2_A_0.1_/_0.4_/_0.8_/_0.2_ hydrogel samples were subjected to a 300 s cyclic test. The strain loading program was as follows: 2% strain for 60 s, followed by 200% strain for 60 s, then repeated sequences of 2% strain for 60 s, 200% strain for 60 s, and finally 2% strain for 60 s. The storage modulus, loss modulus, and loss factor of the samples were recorded throughout the cyclic test. (2) Strain amplitude sweep test: At room temperature and a fixed angular frequency of 10 rad/s, the same hydrogel samples were subjected to a strain amplitude sweep from 0.1% to 200%. The storage modulus, loss modulus, and loss factor were measured.

### 4.7 Lap‐Shear and Vertical Peel Adhesion Tests

Lap‐shear tests were performed using a universal testing machine to evaluate the adhesion properties of PC_0.1_ and PC_0.1_/_0.2_A_0.1_/_0.4_/_0.8_/_0.2_ hydrogels. The sample (10 mm × 10 mm × 1 mm) was placed between two sheets of PET, PDMS, TPU, PTFE, or PE, forming an overlap area of 10 mm × 10 mm. Before testing, a contact pressure of 0.98 N was applied and held for 10 min to ensure good contact. All tests were conducted at room temperature at a shear rate of 10 mm/min. Adhesion tests were performed using a rheometer. The hydrogel (diameter: 20 mm, thickness: 1 mm) was placed on a PDMS or PTFE plate, and an axial force of approximately 0.1 N was applied and held for 10 min. Subsequently, a tensile force was applied vertically at a rate of 10 mm/min at room temperature to separate the plate from the hydrogel. The adhesion strength was calculated as the maximum force divided by the contact area.

### 4.8 SEM Characterization

Gemini SEM 500 field emission scanning electron microscope (ZEISS, Germany) was used for observation, with an accelerating voltage range of 1–5 kV. The PC_0.2_A_0.2_ hydrogel was frozen in liquid nitrogen for 30 min and then transferred to a freeze dryer for 24 h of drying. After drying, the sample was mounted onto either a planar sample stage or a 90° cross‐sectional sample stage using double‐sided conductive tape, sputter‐coated with gold for 30 s, and then imaged by SEM to observe the phase separation of the gel.

### 4.9 Differential Scanning Calorimetry (DSC)

Differential scanning calorimetry (DSC) measurements were performed on PC_0.1_/_0.2_A_0.1_/_0.4_/_0.8_/_0.2_ hydrogel samples using a differential scanning calorimeter (DSC 300, NETZSCH, Germany). Under a nitrogen atmosphere, the samples were heated from 60°C to 180°C at a heating rate of 10 K/min, and aluminum crucibles were used during the test.

### 4.10 Density Functional Theory (DFT) Calculations

First‐principles calculations were performed using the Vienna Ab initio Simulation Package (VASP) based on density functional theory (DFT). The exchange‐correlation functional was described by the Perdew–Burke–Ernzerhof (PBE) functional within the generalized gradient approximation (GGA). The interaction between ionic cores and valence electrons was treated using the projector augmented wave (PAW) method, and the plane‐wave cutoff energy was set to 450 eV. To better describe the weak interactions in the system, DFT‐D3 dispersion correction was applied in all calculations. During structural optimization, the total energy convergence criterion was set to 1.0 × 10^−5^ eV, and the atomic force convergence threshold was set to 0.02 eV·Å^−1^. Brillouin zone integration was performed using a 3 × 3 × 3 Monkhorst–Pack *k*‑point mesh. Unless otherwise specified, all atomic positions were fully relaxed. To investigate the coordination interaction between Ag^+^ in AgNO_3_ and CTBA, two typical coordination models were constructed: (1) a model where Ag^+^ coordinates with the S atom on the thiophene ring, and (2) a model where Ag^+^ coordinates with the carboxylic acid group in a bidentate bridging mode. The binding energy was calculated according to Equation (1):

(1) $$E_{b}=E_{Ag^{+}}+E_{\mathrm{CTBA}}-E_{\mathrm{complex}}$$

$E_{Ag^{+}}$ is the total energy of an isolated Ag^+^, *E*_CTBA_is the total energy of an isolated CTBA molecule, and *E*_complex_ is the total energy of the optimized Ag^+^─CTBA coordination complex. A larger *E*_b_value indicates a stronger interaction between Ag^+^ and the corresponding coordination site. By comparing the binding energies of the Ag^+^─S and Ag^+^─O coordination models, the preferential coordination site of Ag^+^ within the CTBA molecule can be determined, providing a theoretical basis for analyzing the strength of coordination interactions in experiments.

### 4.11 Fourier Transform Infrared Spectrometer (FTIR)

FT‐IR is primarily based on the absorption spectra of chemical bonds and functional groups in a sample at different wavenumbers. It was used to analyze the chemical bonds and functional groups of hydrogels, including PC_0.1_ and PC_0.1_/_0.2_A_0.1_/_0.4_/_0.8_/_0.2_. The measurements were performed using a Fourier transform infrared spectrometer (FT‐IR, model: ALPHA II) manufactured by Bruker Corporation (Germany), with a measurement range of 4000–400 cm^−1^.

## Author Contributions

**Shixia Lan**: methodology, data curation, investigation, writing – original draft, formal analysis. **Lin Zhang**: conceptualization, software, methodology. **Yunyun Dong**: conceptualization, methodology, software. **Yongyun Mao**: writing – review and editing, funding acquisition, project administration, resources, supervision. **Wanbiao Hu**: funding acquisition, writing – review and editing, project administration, resources, supervision.

## Conflicts of Interest

The authors declare no conflicts of interest.

## Supporting information




**Supporting File**: advs77707‐sup‐0001‐SuppMat.docx.

## Data Availability

The data that support the findings of this study are available on request from the corresponding author. The data are not publicly available due to privacy or ethical restrictions.
