# Supplementary material for: Sulfur–Oxygen Competitive Coordination Allosteric Hydrogel for Triboelectric Nanogenerator‐Based Intelligent Tactile Recognition
Source: Adv Sci (Weinh). 2026 Sep 27:e77707. Online ahead of print. doi: 10.1002/advs.77707 (PMC13616259; doi:10.1002/advs.77707)
Supplement: Supplementary file 1 [file ADVS-9999-e77707-s001.docx]

**Supporting Information**

**Sulfur–Oxygen Competitive Coordination Allosteric Hydrogel for Triboelectric Nanogenerator-Based Intelligent Tactile Recognition**

**Shixia Lan^1,3#^, Lin Zhang^4#^, Yunyun Dong^4^*, Yongyun Mao^1^*, Wanbiao Hu^1,2,3^***

^1^Yunnan Key Laboratory of Electromagnetic Materials and Devices, School of Materials and Energy, Yunnan University, Kunming 650091, P. R. China

^2^School of Engineering, Yunnan University, Kunming 650091, P. R. China

^3^Southwest United Graduate School, Kunming 650092, PR China

^4^School of Software and AI, Yunnan University, Kunming 650091, P. R. China

*E-mail: [maoyongyun123@126.com](mailto:maoyongyun123@126.com), [huwanbiao@ynu.edu.cn](mailto:huwanbiao@ynu.edu.cn)

#These authors contributed equally to this work and they should be regarded as co-first authors

**Table S1**. FTIR peak shifts associated with Ag^+^-S coordination and peak positions of symmetric (ν_s_) and asymmetric (ν_as_) stretching vibrations of carboxylate groups in PC_0.1_ and PCA hydrogels with different Ag⁺ contents.

| Sample | C-S bond shift induced by Ag^+^-S coordination/cm^-1^ | ν_as_(coo-) after Ag-O bridged bidentate coordination/cm^-1^ | ν_s_(coo-) after Ag-O bridged bidentate coordination/cm^-1^ | Δν | coordination mode of Ag-O |
| --- | --- | --- | --- | --- | --- |
| PC_0.1_ | 986 | 1587 | 1431 | — | — |
| PC_0.1_A_0.1_ | 973 | 1574 | 1418 | 156 | bridging bidentate coordination |
| PC_0.1_A_0.2_ | 971 | 1582 | 1423 | 159 |  |
| PC_0.1_A_0.3_ | 970 | 1587 | 1428 | 159 |  |
| PC_0.1_A_0.4_ | 971 | 1571 | 1423 | 148 |  |
| PC_0.1_A_0.5_ | 970 | 1579 | 1424 | 155 |  |
| PC_0.1_A_0.6_ | 972 | 1581 | 1430 | 151 |  |
| PC_0.1_A_0.7_ | 968 | 1579 | 1422 | 157 |  |
| PC_0.1_A_0.8_ | 966 | 1571 | 1420 | 151 |  |

As the Ag⁺ content increases, the C‑S characteristic peak continuously shifts from 986 cm⁻¹ to a lower wavenumber (966 cm⁻¹). This redshift can be attributed to the coordination of S with Ag⁺, where the lone pair electrons on sulfur participate in metal–ligand interactions, thereby weakening the C‑S bond strength, which confirms the existence of Ag⁺–S coordination. Meanwhile, the asymmetric and symmetric stretching vibration peaks of the carboxylate group are located at 1571–1587 cm⁻¹ and 1418–1430 cm⁻¹, respectively, and the peak splitting Δν between them remains consistently below 160 cm⁻¹. According to the classical criterion for carboxylate coordination vibrations,^[1, 2]^ this range of Δν is characteristic of bridging bidentate coordination, indicating that Ag⁺ can further form bridging structures with carboxylate groups. Collectively, these results demonstrate that Ag⁺ participates in both Ag⁺–S and Ag⁺–O coordination in the system, and that the different dominant coordination structures formed at different ratios regulate the evolution of the network structure.

**Table S2.** EXAFS fitting parameters at the Ag K-edge for Ag foil and samples with Ag⁺:S = 1 and Ag⁺:S > 1 (S₀² = 0.85, determined from Ag foil).

|  | shell | CN*^a^* | R*^b^*(Å) | σ^2^*^c^*(Å^2^) | ΔE_0_*^d^*(eV) | R factor |
| --- | --- | --- | --- | --- | --- | --- |
| Ag-foil | Ag-Ag | 12.0 | 2.86±0.01 | 0.0098 | 2.3±0.3 | 0.0027 |
| Ag⁺:S = 1 | Ag-S1 | 0.7±0.3 | 2.12±0.01 | 0.0030 | -9.0±1.2 | 0.0163 |
|  | Ag-S2 | 1.3±0.1 | 2.60±0.01 | 0.0037 |  |  |
| Ag⁺:S > 1 | Ag-O | 2.0 | 2.03±0.01 | 0.0011 | -0.8±26 | 0.0151 |
|  | Ag-S | 1.8±0.3 | 2.59±0.01 | 0.0032 |  |  |
|  | Ag-Ag | 1.8±0.4 | 3.19±0.01 | 0.0055 |  |  |

*^a^CN*: coordination numbers; *^b^R*: bond distance; *^c^σ*^2^: Debye-Waller factors; *^d^* Δ*E*_0_: the inner potential correction. R factor: goodness of fit. Error bounds that characterize the structural parameters obtained by EXAFS spectroscopy were estimated as CN ± 20%; R ± 1%; σ^2^ ± 20%.

Under the condition Ag⁺:S = 1, only Ag⁺–S coordination paths are fitted around Ag⁺, with bond lengths of 2.12 Å and 2.60 Å, respectively, and a total coordination number of approximately 2. No Ag⁺–Ag⁺ path is observed, indicating that silver ions mainly reside in a dispersed local coordination environment dominated by Ag⁺–S. Under the condition Ag⁺:S > 1, in addition to the Ag⁺–S path, a shorter Ag⁺–O coordination (2.03 Å) and an Ag⁺–Ag⁺ path (3.19 Å) also appear, demonstrating that as the Ag⁺ content increases, the local coordination environment gradually evolves from a single Ag⁺–S structure to a multi‑path complex structure where Ag⁺–S, Ag⁺–O, and Ag⁺–Ag⁺ coexist. The coordination number of the Ag⁺–O bond is 2. Combined with the carboxylate vibration peak splitting Δν = 148–159 cm⁻¹ from FTIR, it can be inferred that this coordination conforms to a bridging bidentate mode, and the Ag⁺–Ag⁺ path likely arises from the two silver ion centers within the bridging ligand. The existence of the Ag–S coordination path is also consistent with the shift of the C‑S peak in the FTIR measurements (Table S1), confirming that the Ag–S coordination structure still exists in the excess‑silver system, albeit not as the dominant species.

**Table S3.** Detailed compositions of PC, PA, PCA hydrogels, and CTBA/Ag^+^ molar ratios of PCA hydrogels.

| Hydrogel | AM (wt%) | MBAA (wt%) | NMA (wt%) | APS (wt%) | CTBA  (wt%) (g) | | AgNO_3_  (wt%) (g) | | Nominal CTBA concentration (mol) | Nominal AgNO_3_ concentration (mol) | Nominal CTBA/Ag+ molar ratio |
| --- | --- | --- | --- | --- | --- | --- | --- | --- | --- | --- | --- |
| PC_0.1_ | 14.6022 | 0.1565 | 0.1565 | 0.2086 | 1.4349 | 0.0688 | — | — | 0.0004 | — | — |
| PA_0.1_ | 14.6048 | 0.1565 | 0.1565 | 0.2086 | — | — | 1.42 | 0.0679 | — | 0.0004 | — |
| PC_0.1_A_0.1_ | 14.3981 | 0.1543 | 0.1543 | 0.2057 | 1.4149 | 0.0688 | 1.39 | 0.0679 | 0.0004 | 0.0004 | 1 : 1 |
| PC_0.1_A_0.4_ | 13.8188 | 0.1481 | 0.1481 | 0.1974 | 1.3579 | 0.0688 | 5.36 | 0.2718 | 0.0004 | 0.0016 | 1 : 4 |
| PC_0.1_A_0.8_ | 13.1151 | 0.1405 | 0.1405 | 0.1874 | 1.2888 | 0.0688 | 10.2 | 0.5436 | 0.0004 | 0.0032 | 1 : 8 |
| PC_0.2_A_0.2_ | 14.0043 | 0.1500 | 0.1500 | 0.2001 | 2.7524 | 0.1376 | 2.72 | 0.1359 | 0.0008 | 0.0008 | 1 : 1 |

**Note:** The nominal molar concentrations were calculated based on 4 mL of solvent, whereas the weight fractions were calculated relative to the total mass of the precursor formulation. **In the notation PC*_x_*A*_y_*, *x* and *y* denote the molar ratios of CTBA and AgNO_3_, respectively, calculated based on 4 mL of solvent. Accordingly, PC_0.1_A_0.1_, PC_0.1_A_0.4_, PC_0.1_A_0.8_, and PC_0.2_A_0.2_ correspond to nominal** CTBA/Ag^+^ **molar ratios of 1:1, 1:4, 1:8, and 1:1, respectively.**

The nominal molar amounts were calculated based on 4 mL of solvent, whereas the weight fractions were calculated relative to the total mass of the precursor formulation, including the solvent and all added components. For example, for PC0.1, the nominal amount of CTBA was calculated as nCTBA = c V = 0.1 mol L−1 × 0.004 L = 0.0004 mol, corresponding to a CTBA mass of mCTBA = n M = 0.0004 mol × 171.97 g mol−1 ≈ 0.0688 g. Taking 4 mL of solvent as approximately 4.0000 g, together with 0.7000 g AM, 0.0075 g MBAA, 0.0075 g NMA, and 0.0100 g APS, the total precursor mass was 4.793788 g. Therefore, the CTBA weight fraction was calculated as 0.068788 / 4.793788 × 100% = 1.4349 wt%.

**Figure S1. Chemical structure of the PCA hydrogel network at different Ag^+^ -S (CTBA) ratios: (a) Ag^+^: CTBA =1, (b)** **Ag^+^: CTBA >1.**

When the coordination configuration is the “soft sulfur‑philic (Ag⁺–S)” mode, it is defined as the softened state (or soft and highly adhesive state), indicating that under this coordination, the interchain crosslinks within the network are weakened, the network becomes discrete, the structure softens, and the hydrogel exhibits a softened state. When the coordination configuration is the “hard oxygen‑philic (Ag⁺–O)” mode, it is defined as the reinforced state (or enhanced mechanical state), indicating that Ag⁺ forms stable bridging bidentate ligands with carboxylate groups, the network re‑establishes a more continuous and robust physical crosslinking, and the hydrogel exhibits a reinforced state.

**Figure S2. Schematic illustration of the reversible structural transformation of the PCA hydrogel network.**

Starting from the PC_0.1_ base hydrogel system, upon addition of Ag⁺ until approaching the equimolar ratio, the sulfur on the thiophene ring forms a coordination structure with Ag⁺ through σ–π interactions, and the network enters a softened state. Upon further addition of Ag⁺, Ag⁺ forms stable bridging bidentate ligands with carboxylate groups, and the network enters a reinforced state. If CTBA is then added again, the gel system returns to the equimolar coordination state, forming PC_0.2_A_0.2_. However, due to the further increase in Ag–S coordination content, phase separation is induced, causing the gel to soften once more. This reflects the network structural evolution during the "reinforcement – softening – re‑reinforcement – re‑softening" process of the hydrogel.

**Figure S3. (a) FTIR spectra of PC hydrogels with different CTBA contents. (b) FTIR spectra of** **PC_0.1_ and PCA hydrogels with different Ag⁺ contents.**

The shift of the C–S peak and the frequency difference between the two carboxylate peaks (detailed results are presented in Table S1) indicate the existence of both Ag⁺–S and Ag⁺–O coordinations.

**Figure S4. Synchrotron X-ray absorption spectroscopy analysis of Ag^＋^-S coordination structures. (a) R-space fitting, (b) k-space fitting. (c) wavelet transform (WT) 3D spectra of the equimolar Ag⁺-S coordination system (Ag⁺:S=1). (d) R-space fitting, (e) k-space fitting, and (f) wavelet transform (WT) 3D spectra of the excess Ag⁺ coordination system (Ag⁺:S > 1).**

Figure S4a shows the R‑space fitting plot for the Ag⁺:S = 1 sample. According to the results in Table S2, no Ag⁺–Ag⁺ related path is formed; only the Ag⁺–S path is present. This indicates that Ag⁺ is mainly captured by sulfur‑containing sites, forming an S‑dominated, low‑coordination, locally dispersed coordination environment. Figure S4b shows the k‑space fitting plot for the Ag⁺:S = 1 sample. The k‑space oscillations are smooth and of relatively low frequency, consistent with the scattering characteristics dominated by heavy scatterers. Moreover, the fitting curve reproduces the experimental curve well, confirming that no additional Ag⁺–O or Ag⁺–Ag⁺ paths need to be introduced, and the local coordination environment around Ag⁺ is predominantly Ag⁺–S. Figure S4c presents the three‑dimensional wavelet transform plot corresponding to Figure 3b. The intensity distribution in the plot is concentrated, indicating that the local coordination environment around Ag⁺ in the equimolar system is relatively simple and dominated by a single main scattering path. This is consistent with the Ag⁺–S path and the absence of a significant Ag⁺–Ag⁺ path obtained from Table S2, confirming that silver mainly resides in a dispersed local coordination environment dominated by Ag⁺–S.

Figure S4d shows the R‑space fitting plot for the Ag⁺:S > 1 sample (corresponding to Figure 3d). Combined with the fitting results in Table S2, it indicates that Ag⁺–O and Ag⁺–Ag⁺ paths appear in this system, suggesting the formation of bridging coordination and a more complex structure. Figure S4e presents the k‑space fitting plot for the Ag⁺:S > 1 sample (corresponding to Figure 3e). The higher‑frequency oscillations indicate that the Ag⁺ coordination paths are no longer singular and the local structure is more complex. Figure S4f shows the three‑dimensional wavelet transform plot corresponding to Figure 3c. Compared with the equimolar system, the Ag⁺‑excess system exhibits a broader and more complex intensity distribution, indicating that the local coordination environment around silver evolves from a relatively simple S‑dominated path to a multi‑coordination complex structure involving Ag⁺–O and Ag⁺–Ag⁺ related paths.

**Figure S5. (a–d) Photographs illustrating the macroscopic changes of the hydrogel before and after Ag^+^-S coordination. (e) Macroscopic phase separation observed with increasing equimolar** **Ag^+^-S coordination content.**

When CTBA or Ag⁺ was added alone to the PAM hydrogel, the resulting hydrogel macroscopically maintained a certain strength and shape under stretching (Figure S5 b‑c). However, when both were present at an equimolar ratio, the gel no longer retained similar strength under the effect of silver‑sulfur coordination and exhibited a softened state upon stretching (Figure 3f). When excess silver ions were added to PC_0.1_A_0.4_, as seen in Figure S5d and Figure 3f, the gel entered a reinforced state under the action of bridging bidentate ligands, recovering a certain strength and maintaining its original shape during stretching. When the equimolar ratio was re‑established to PC_0.2_A_0.2_, the macroscopic shape of the gel was similar to that of PC_0.1_A_0.1_, and the gel deformed upon stretching, presenting a softened state (Figure 3g). Moreover, under equimolar silver‑sulfur coordination, the PC_0.1_A_0.1_ system solution began to show turbidity compared to the clear PAM solution (Figure S5e), indicating that silver‑sulfur coordination formed enriched domains, i.e., microphase separation. The PC_0.2_A_0.2_ system solution exhibited even more pronounced turbidity, indicating that the phase separation further evolved from microphase to larger heterogeneous phase domains. This change is the primary cause of the macroscopic transformation of the gel.

**Figure S6. Elemental mapping of (a–d) PAM hydrogel.**

Figure S6 shows the elemental mappings of C, N, and O in the PAM hydrogel, which exhibit a uniform microstructure and homogeneous elemental distribution. In contrast, compared with the elemental mappings of C, Ag⁺, and S in PC_0.2_A_0.2_ (Figures 3h–k), the PCA gel displays a heterogeneous microstructure, which is attributed to phase separation induced by the enrichment of Ag⁺–S coordination.

**Figure S7. Hofmeister effect on hydrogel mechanical performance.**

Figure S7 compares the effects of NaNO₃ and AgNO₃ on the mechanical properties of the gel; the test results rule out the possibility that the NO₃⁻ ion (i.e., the Hofmeister effect) causes a decrease in gel strength. Based on the above test results, it is concluded that the changes in the mechanical properties of the PCA gel (specifically the appearance of the softened state) originate primarily from the Ag⁺–S dynamic coordination structure.

**Figure S8. (a) Digital photos of tensile tests of PC_0.1_ and PCA hydrogels with different Ag⁺ contents. The load applied to PC_0.2_A_0.2_ is 5 g due to its limited tensile strength, while 20 g loads are applied to the other samples. (b) Photographs showing the tensile deformation behavior of PC_0.1_ and PC_0.2_A_0.2_ hydrogels under different strain levels.**

Figure S8 shows photographs corresponding to the mechanical performance data in Figure 4c. Figure S8aⅰ-ⅴ presents tensile photographs of PC_0.1_ and various PCA samples. Notably, PC_0.2_A_0.2_ can only bear a 5 g weight (a-ⅴ), indicating that under the effect of Ag⁺–S coordination, the gel network becomes looser and exhibits limited strength. Figure S8bⅰ-ⅴ compares the deformation photographs of PC_0.1_ and PC_0.2_A_0.2_ under different strains, demonstrating that PC_0.2_A_0.2_ is softer and more prone to large deformation.

**Figure S9. (a–d) Self-healing process of PC_0.1_ hydrogel. (e–j) Self-healing process and corresponding tensile photographs of PCA hydrogels with two different Ag⁺ contents.**

Figure S9 provides photographic evidence complementary to the tensile behavior and self-healing efficiency of the hydrogels after healing, as presented in Figures 4d–e. Figures S9a–d show the cutting, reconnection, healing, and subsequent stretching processes of two PC_0.1_ hydrogel pieces, one transparent and the other dyed, demonstrating reformation of the fractured interface and partial recovery of tensile deformability. Figures S9e–j further illustrate the self-healing behavior of PC_0.1_A_0.1_ and PC_0.1_A_0.8_ hydrogels. After the two hydrogels were separately cut, the resulting sections were cross-matched and brought into contact, allowing an integrated hydrogel structure to be re-established. As discussed in the main text, PC_0.1_A_0.1_ exhibits relatively high segmental mobility, and Ag+–S dynamic coordination plays a key role in the interfacial reassembly process, facilitating reconstruction of the fractured interface. Therefore, even when coupled with the more rigid PC_0.1_A_0.8_ network, a stable healed interface can still be formed through interfacial reassembly. The healed sample remained intact without obvious cracking or interfacial separation upon sequential loading with 1, 10, and 20 g weights, further supporting the effective interfacial reassembly associated with Ag+–S dynamic coordination and confirming that the healed interface retained good structural integrity and load-bearing capability.

**Figure S10. (a–d) Lap-shear tests of PC_0.1_, PC_0.1_A_0.1_, PC_0.1_A_0.4_, PC_0.1_A_0.8_ hydrogels against various triboelectric materials. (e–h) Photographs illustrating the adhesion behavior of PC_0.1_ and PC_0.2_A_0.2_ hydrogels on different triboelectric materials, including PET (e-f), TPU (g-h) and PE (i-j).**

Figures S10a–d present the lap‑shear test results of PC_0.1_, PC_0.1_A_0.1_, PC_0.1_A_0.4_, and PC_0.1_A_0.8_ gels against different tribological materials. The results demonstrate that the softened state of the PCA hydrogel is not only a rheological characteristic of the internal network but is also reflected in the interfacial adhesion and peeling dissipation capacity when interacting with external materials. A larger strain extension in the test indicates that the system can withstand greater deformation before debonding, implying stronger interfacial energy dissipation capability. From Figures S10a–d, it can be seen that all gels exhibit a large strain on PDMS and the smallest strain on PTFE, with PC₀.₁A₀.₁ showing the most pronounced strain behavior. This confirms that under coordination effects, interfacial rearrangement occurs more readily, facilitating extension and dissipation during shearing. Compared with the softened state of PC_0.1_A_0.1_, the networks of PC_0.1_A_0.4_ and PC_0.1_A_0.8_ become more continuous again under bridging reinforcement, resulting in reduced strain variation on different materials. However, at the same time, the interfacial shear stress is relatively higher, and the peak stress is elevated, indicating that the gel has entered a reinforced state. Figures S10e–j show actual adhesion photographs of PC_0.1_ and PC_0.2_A_0.2_ on PET, TPU, and PE substrates. It can be clearly seen that PC_0.2_A_0.2_ exhibits more pronounced interfacial tensile deformation and morphological extension during peeling, indicating that it more easily forms fully conformal contact on different substrate surfaces and generates greater interfacial energy dissipation. This result is consistent with its softened state characteristics observed in rheological tests, enabling the gel to exhibit stronger interfacial adaptability in subsequent triboelectric nanogenerator performance output.

**Figure S11. (a–l) Output performance (current, voltage, and transferred charge) of TENG devices based on PC_0.1_ and PCA hydrogels with different Ag⁺ contents, paired with various triboelectric materials, including PTFE, PET, TPU and PE.**

Figure S11 illustrates the output performance of gels with different concentrations against various triboelectric materials. Similar to the triboelectric output performance with PDMS, the gels also exhibit the following trend in tests with the other four materials: under equimolar Ag⁺–S coordination, the softened state of the gel enables better conformal contact with different triboelectric materials, resulting in higher triboelectric output. However, when the Ag⁺ content further increases, the gel transitions to a reinforced state, and the dominant factor affecting the output gradually shifts from "adhesion" to "difference in electron affinity", leading to a decrease in triboelectric output. Thus, different Ag⁺ coordination structures not only alter the mechanical properties of the gel but also affect the interfacial charge transfer behavior of the device.

**Figure S12. (a) Charging time curves of capacitors with different capacitance values (0.22, 0.33, 0.47, 1.0 and 2.2 μF). (b) Output voltage and current of TENG loaded with different external resistances. (c) Dependence of peak power density on the external load resistances. (d–i) Real-time voltage signals of the TENG in various application scenarios.**

The triboelectric nanogenerator test results in Figure S12 show that PC_0.2_A_0.2_ can not only output signals but also charge capacitors and match the load. When the load resistance is 500 MΩ, the device achieves a maximum power density of approximately 183 nW cm⁻², and it exhibits excellent triboelectric sensing performance under different wearing motions.

**Figure S13. (a) Output signals of hydrogels with Ag⁺:S > 1 during finger bending. (b) Output signals of hydrogels with Ag⁺:S = 1 during finger bending.**

Under the condition of Ag⁺:S > 1 (Figure S13a), the output electrical signal of the hydrogel during finger bending is weaker than that under Ag⁺:S = 1. This is because the hydrogel's weak interfacial adhesion and electrical output capability limit the bending range of the robotic hand. In contrast, under the condition of Ag⁺:S = 1, during finger bending, the Ag⁺-S coordination optimizes the hydrogel network structure and improves its interfacial adhesion, thereby enhancing the interfacial charge transfer efficiency and electrical output capability. As a result, the electrical signal amplitude is significantly increased, enabling a larger range of bending signal output from the robotic hand (Figure S13b).

**Figure S14. Output signal responses of the hydrogel integrated into the smart glove as an electronic skin during tactile recognition of different materials.**

When the hydrogel is integrated into a smart glove as an electronic skin for tactile recognition, its electrical signal responses to different materials, compared with experimental test results, show that under actual wearing conditions, the signal fluctuations for different materials become similar, leading to poor material discrimination ability.

**Figure S15. (a) Machine recognition accuracy before model training: 20.32%. (b) Recognition accuracy of the sensor glove in actual wearing tests before model training: 20.97%.**

Before model training, direct prediction evaluation was performed using 1000 machine test samples (Figure S15a) and 350 gel sensor glove wearing test samples (Figure S15b). The results show that under both conditions, the confusion matrices exhibit no obvious diagonal clustering, and extensive cross‑misclassification occurs among different materials. The corresponding prediction accuracies are only 20.32% and 20.97%, respectively, both close to the theoretical random guessing value of 20% for five‑class classification. This indicates that the model in its randomly initialized state has not yet learned effective features to distinguish different materials. It should be noted that although actual wearing conditions typically introduce complex factors such as interfacial contact fluctuations, motion disturbances, and environmental noise, the recognition performance under both test scenarios remains at a level close to random guessing based on the current untrained results. Therefore, subsequent introduction of feature extraction and training algorithms is necessary to improve material recognition accuracy.

1 Y. W. Sen Lin, Ruihong Deng, Qianyong Cao, **2015**, 32, 1114.

2 P. Uznanski, J. Zakrzewska, F. Favier, S. Kazmierski, E. Bryszewska, *Journal of Nanoparticle Research* **2017**, 19.
